# Supplementary material for: Oviposition behaviour of mated or unmated Cleruchoides noackae (Hymenoptera: Mymaridae)
Source: PLoS One. 2020 Oct 2;15(10):e0239285. doi: 10.1371/journal.pone.0239285 (PMC7531830; doi:10.1371/journal.pone.0239285)
Supplement: Attachment — Submitted filename: PONE-D-20-06870_reviewer.pdf [file pone.0239285.s005.pdf]

PLOS ONE

Oviposition behaviour of mated or unmated Cleruchoides noackae (Hymenoptera: Mymaridae) females

--Manuscript Draft--

**Manuscript Number:**	PONE-D-20-06870
**Article Type:**	Research Article
**Full Title:**	Oviposition behaviour of mated or unmated Cleruchoides noackae (Hymenoptera: Mymaridae) females
**Short Title:**	Ovipostion behaviour of Cleruchoides noackae females
**Corresponding Author:**	Luciane Katarine Becchi, Msc<br>Universidade Estadual Paulista Julio de Mesquita Filho<br>Botucatu, São Paulo BRAZIL
**Keywords:**	biological control; courtship behaviour; egg parasitoid; oviposition behaviour
**Abstract:**	Cleruchoides noackae (Hymenoptera: Mymaridae), native to Australia, is the most promising biological control agent for Thaumastocoris peregrinus (Hemiptera: Thaumastocoridae), an exotic Eucalyptus spp. pest. The objective was to study the courtship behaviour, mating and oviposition of virgin or mated C. noackae females parasitizing T. peregrinus eggs. The virgin or mated females were placed individually in polystyrene flasks with 10 T. peregrinus eggs each, and the oviposition behaviour, percentage of eggs inserted and parasitized, viability and sex ratio of C. noackae were recorded. This species lacked defined courtship behaviour and mated in less than an hour after emergence. The time spent finding the first host, evaluating the host and inserting the ovipositor and the percentage of inserted and parasitized eggs and their viability were similar for mated and unmated C. noackae females. The time taken to locate other host eggs and the sex ratio (female: male) of the offspring were higher for mated females. The ability of C. noackae to mate and oviposit in less than 1 hour and the parasitism of T. peregrinus eggs by virgin and mated females can improve breeding and biological control of T. peregrinus.
**Order of Authors:**	Luciane Katarine Becchi, Msc
	Carolina Jorge
	Gabriella Ferreira de Camargo
	Leonardo Rodrigues Barbosa
	Marcus Alvarenga Soares
	José Eduardo Serrão
	José Cola Zanuncio
	Carlos Frederico Wilcken
**Additional Information:**	
**Question**	**Response**
**Financial Disclosure**<br><br>Enter a financial disclosure statement that describes the sources of funding for the work included in this submission. Review the submission guidelines for detailed requirements. View published research articles from *PLOS ONE* for specific examples.	This research was funded through “Coordenação de Aperfeiçoamento de Pessoal de Nível Superior (CAPES)”, “Conselho Nacional de Desenvolvimento Científico e Tecnológico (CNPq)”, “Fundação de Amparo à Pesquisa do Estado de Minas Gerais (FAPEMIG)” and “Programa Cooperativo sobre Proteção Florestal (PROTEF)”. The funders had no role in study design, data collection and analysis, decision to publish, or preparation of the manuscript.

Powered by Editorial Manager® and ProduXion Manager® from Aries Systems Corporation

This statement is required for submission and **will appear in the published article** if the submission is accepted. Please make sure it is accurate.<br><br>**Unfunded studies**<br>Enter: *The author(s) received no specific funding for this work.*<br><br>**Funded studies**<br>Enter a statement with the following details:<br>• Initials of the authors who received each award<br>• Grant numbers awarded to each author<br>• The full name of each funder<br>• URL of each funder website<br>• Did the sponsors or funders play any role in the study design, data collection and analysis, decision to publish, or preparation of the manuscript?<br>• **NO** - Include this sentence at the end of your statement: *The funders had no role in study design, data collection and analysis, decision to publish, or preparation of the manuscript.*<br>• **YES** - Specify the role(s) played.<br><br>* typeset	
**Competing Interests**<br><br>Use the instructions below to enter a competing interest statement for this submission. On behalf of all authors, disclose any competing interests that could be perceived to bias this work—acknowledging all financial support and any other relevant financial or non-financial competing interests.<br><br>This statement **will appear in the published article** if the submission is accepted. Please make sure it is accurate. View published research articles from *PLOS ONE* for specific examples.	The authors have declared that no competing interests exist.

**NO authors have competing interests**<br><br>Enter: *The authors have declared that no competing interests exist.*<br><br>**Authors with competing interests**<br><br>Enter competing interest details beginning with this statement:<br><br>*I have read the journal's policy and the authors of this manuscript have the following competing interests: [insert competing interests here]*<br><br>* typeset	
**Ethics Statement**<br><br>Enter an ethics statement for this submission. This statement is required if the study involved:<br><br>• Human participants<br>• Human specimens or tissue<br>• Vertebrate animals or cephalopods<br>• Vertebrate embryos or tissues<br>• Field research<br><br>Write "N/A" if the submission does not require an ethics statement.<br><br>General guidance is provided below. Consult the submission guidelines for detailed instructions. **Make sure that all information entered here is included in the Methods section of the manuscript.**	N/A

Powered by Editorial Manager® and ProduXion Manager® from Aries Systems Corporation

**Format for specific study types**<br><br>**Human Subject Research (involving human participants and/or tissue)**<br>• Give the name of the institutional review board or ethics committee that approved the study<br>• Include the approval number and/or a statement indicating approval of this research<br>• Indicate the form of consent obtained (written/oral) or the reason that consent was not obtained (e.g. the data were analyzed anonymously)<br><br>**Animal Research (involving vertebrate animals, embryos or tissues)**<br>• Provide the name of the Institutional Animal Care and Use Committee (IACUC) or other relevant ethics board that reviewed the study protocol, and indicate whether they approved this research or granted a formal waiver of ethical approval<br>• Include an approval number if one was obtained<br>• If the study involved *non-human primates*, add *additional details* about animal welfare and steps taken to ameliorate suffering<br>• If anesthesia, euthanasia, or any kind of animal sacrifice is part of the study, include briefly which substances and/or methods were applied<br><br>**Field Research**<br><br>Include the following details if this study involves the collection of plant, animal, or other materials from a natural setting:<br>• Field permit number<br>• Name of the institution or relevant body that granted permission	
**Data Availability**<br><br>Authors are required to make all data underlying the findings described fully available, without restriction, and from the time of publication. PLOS allows rare exceptions to address legal and ethical concerns. See the PLOS Data Policy and FAQ for detailed information.	Yes - all data are fully available without restriction

A Data Availability Statement describing where the data can be found is required at submission. Your answers to this question constitute the Data Availability Statement and **will be published in the article**, if accepted.<br><br>**Important:** Stating 'data available on request from the author' is not sufficient. If your data are only available upon request, select 'No' for the first question and explain your exceptional situation in the text box.<br><br>Do the authors confirm that all data underlying the findings described in their manuscript are fully available without restriction?	
**Describe where the data may be found in full sentences. If you are copying our sample text, replace any instances of XXX with the appropriate details.**<br><br>• If the data are **held or will be held in a public repository**, include URLs, accession numbers or DOIs. If this information will only be available after acceptance, indicate this by ticking the box below. For example: *All XXX files are available from the XXX database (accession number(s) XXX, XXX.*).<br>• If the data are all contained **within the manuscript and/or Supporting Information files**, enter the following: *All relevant data are within the manuscript and its Supporting Information files.*<br>• If neither of these applies but you are able to provide **details of access elsewhere**, with or without limitations, please do so. For example:<br><br>*Data cannot be shared publicly because of [XXX]. Data are available from the XXX Institutional Data Access / Ethics Committee (contact via XXX) for researchers who meet the criteria for access to confidential data.*<br><br>*The data underlying the results presented in the study are available from (include the name of the third party*	All relevant data are within the manuscript and its Supporting Information files.

and contact information or URL).<br>• This text is appropriate if the data are owned by a third party and authors do not have permission to share the data.<br><br>* typeset	
Additional data availability information:	Tick here if your circumstances are not covered by the questions above and you need the journal’s help to make your data available.

Manuscript

Click here to access/download;Manuscript;Manunscript.Oviposition behaviour

Oviposition behaviour of mated or unmated *Cleruchoides noackae* (Hymenoptera: Mymaridae) females

Luciane Katarine Becchi[1¶*], Carolina Jorge[2¶], Gabriella Ferreira de Camargo[3&], Leonardo Rodrigues Barbosa[4¶], Marcus Alvarenga Soares[5&], José Eduardo Serrão[6&], José Cola Zanuncio[7¶], Carlos Frederico Wilcken[1¶]

[1]Departamento de Proteção Vegetal, Faculdade de Ciências Agronômicas, Universidade Estadual Paulista (UNESP), Campus de Botucatu, Botucatu, São Paulo, Brasil.

[2]Instituto Superior de Estudios Forestales. CENUR Noreste Sede Tacuarembó, Universidad de la República, Tacuarembó, Uruguay.

[3]Suzano Papel e Celulose - Unidade Mucuri. Mucuri, Bahia, Brasil.

[4]Empresa Brasileira de Pesquisa Agropecuária–Embrapa Florestas, Colombo, Paraná, Brasil.

[5]Programa de Pós-Graduação em Produção Vegetal, Universidade Federal dos Vales Jequitinhonha e Mucuri (UFVJM), Diamantina, Minas Gerais, Brasil.

[6]Departamento de Biologia Geral/BIOAGRO, Universidade Federal de Viçosa, Viçosa, Minas Gerais, Brasil.

[7]Departamento de Entomologia/BIOAGRO, Universidade Federal de Viçosa, Viçosa, Minas Gerais, Brasil.

* Corresponding author

E-mail: becchiluciane@gmail.com (LKB)

[¶]These authors contributed equally to this work.

[&]These authors also contributed equally to this work.

Abstract

Cleruchoides noackae (Hymenoptera: Mymaridae), native to Australia, is the most promising biological control agent for *Thaumastocoris peregrinus* (Hemiptera: Thaumastocoridae), an exotic *Eucalyptus* spp. pest. The objective was to study the courtship behaviour, mating and oviposition of virgin or mated *C. noackae* females parasitizing *T. peregrinus* eggs. The virgin or mated females were placed individually in polystyrene flasks with 10 *T. peregrinus* eggs each, and the oviposition behaviour, percentage of eggs inserted and parasitized, viability and sex ratio of *C. noackae* were recorded. This species lacked defined courtship behaviour and mated in less than an hour after emergence. The time spent finding the first host, evaluating the host and inserting the ovipositor and the percentage of inserted and parasitized eggs and their viability were similar for mated and unmated *C. noackae* females. The time taken to locate other host eggs and the sex ratio (female: male) of the offspring were higher for mated females. The ability of *C. noackae* to mate and oviposit in less than 1 hour and the parasitism of *T. peregrinus* eggs by virgin and mated females can improve breeding and biological control of *T. peregrinus*.

Introduction

Australian exotic pest insects damage forest plantations in South America, especially those of the genus *Eucalyptus* (Myrtaceae) [1,2,3]. The bronze bug, *Thaumastocoris peregrinus* Carpintero & Dellapé, 2006 (Hemiptera: Thaumastocoridae), detected in Brazil in 2008 in São Paulo and Rio Grande do Sul states, has spread rapidly

throughout the country [4,5,6]. This exotic insect pest of *Eucalyptus* has a gregarious and sucking habit and damages eucalyptus trees [7,8]. Symptoms caused by this pest include silvering, tanning and drying of leaves due to sap sucking, followed by defoliation of susceptible genotypes [9,4,10]. In addition, reduction in tree diameter, height and volume, besides losses in eucalyptus wood yield of up to 10%-15% were recorded in eucalyptus plantations infested by *T. peregrinus* [4, 3].

Thaumastocoris peregrinus is monitored by sampling the leaves from the middle third on the east side of the eucalyptus plants, counting nymphs, adults and/or eggs of this pest [11] or by using yellow sticky traps [12].

Biological control is the most suitable strategy for *T. peregrinus* management in eucalyptus plantations [12], due to certification requirements for planted areas, lower environmental risks and the high cost of chemical products [13, 14]. Entomopathogenic fungi cause high *T. peregrinus* nymph and adult mortality in the laboratory and also in epizooties in the field [15,16,17]. Native predatory insects such as *Chrysoperla externa* Hagen (Neuroptera: Chrysopidae) [18], *Supputius cincticeps* Stal (Heteroptera: Pentatomidae) [19] and *Atopozelus opsimus* Elkins (Hemiptera: Reduviidae) [20] prey on nymphs and adults of *T. peregrinus* in Brazil. The egg parasitoid, *Cleruchoides noackae* Lin & Huber, 2007 (Hymenoptera: Mymaridae), the main biological control agent of the bronze bug [21,22,14], was introduced to Brazil in 2012 to classical biological control of this pest [6].

Cleruchoides noackae is a solitary endoparasitoid, approximately 0.5 mm long [22], and parasitizes *T. peregrinus* eggs from zero to three days old with an emergence percentage above 60% [6]. The parasitism of *T. peregrinus* by *C. noackae*, in the laboratory

and the field, is 50%-60% [23] and its release in eucalyptus plantations in Brazil reduced the infestation by this pest [3].

Cleruchoides noackae reproduce by arrhenotokous parthenogenesis, with fertilized eggs giving females and unfertilized eggs giving males [24]. In the field, the sex ratio (female: male) of this parasitoid, emerging from *T. peregrinus* eggs, was 0.65 [23]. *Cleruchoides noackae* reared in the laboratory has a short longevity, 1.1 to 3.6 days without and with food, respectively [25]. The reproductive behaviour of *C. noackae* needs further studies, and it is not known when males mate with females in flasks that have been using in the laboratory to rear this parasitoid.

Females of egg parasitoids exhibit distinct oviposition behaviour, consisting of host location and evaluation, ovipositor insertion, host acceptance, oviposition and chemical or mechanical marking to avoid superparasitism [26]. These patterns may be inborn to the species or learned by experience during the host evaluation and oviposition process [27]. The female can, by learning, locate and parasitize the host more efficiently and quickly [28,29]. Therefore, the mating behaviour and oviposition patterns of *C. noackae* should be studied for the purposes of cultivating this parasitoid in the laboratory and increasing its efficiency in the field.

The objective was to evaluate, in the laboratory, the courtship, mating and oviposition behaviour of virgin or mated *C. noackae* females in *T. peregrinus* eggs.

Materials and Methods

Study site

The bioassays were carried out at the Laboratory of Biological Control of Forest Pests (LCBPF) of the São Paulo State University "Júlio de Mesquita Filho" (UNESP), School of Agricultural Sciences (FCA), Botucatu Campus in Botucatu, São Paulo, Brazil.

Host *Thaumastocoris peregrinus*

Thaumastocoris peregrinus eggs were obtained from LCBPF breeding stocks, which were started with eggs, nymphs and adults collected in eucalyptus plantations in the state of Minas Gerais, Brazil. The insects were kept on 3-year-old *Eucalyptus urophylla* var. *platyphylla* (Myrtaceae) branches in 250-ml Erlenmeyer flasks with water in a rectangular plastic tray (40 cm long x 35 cm wide x 8 cm high). Strips of paper towel (1.5 cm wide x 15.0 cm in length) were arranged on the upper surfaces of the branches as a substrate for *T. peregrinus* oviposition for 24 hours. These eggs were used for parasitoid multiplication and experimentation. *Thaumastocoris peregrinus* were reared in an air-conditioned room at 24 ± 2 ºC, 60 ± 10% RH and with a 12-h photophase [30].

Parasitoid *Cleruchoides noackae*

Cleruchoides noackae adults, from the LCBPF stock rearing, started with *T. peregrinus* parasitized eggs collected in eucalyptus plantations in the state of Rio Grande do Sul, Brazil and were kept in transparent polystyrene bottles (7.5 cm high x 3.0 cm in diameter) with paper filter strips (7.0 cm high x 1.5 cm wide) moistened in 50% honey solution as food. Twenty-four-hour-old *T. peregrinus* eggs were offered to the females of this parasitoid in climatic chamber at 24 ± 2 ºC, 60 ± 10% RH and with a 12-h photophase [31].

Courtship and mating behaviour

One hundred *T. peregrinus* eggs, parasitized by *C. noackae*, were stored individually in transparent polystyrene vials (3.5 cm high x 2.0 cm in diameter) at a temperature of 24 ± 2 ºC; RH of 60 ± 10% and with a photophase of 12 h. The adult parasitoids were sexed, shortly after emergence (< 3 hours old), based on the morphology of their antennae: filiform (males) and clavate (female) [22] and received a 50% honey solution. The behaviour, before and during mating, of 11 *C. noackae* virgin couples, placed individually in transparent polystyrene vials (7.5 cm high x 3.0 cm in diameter), was observed for 1 hour under a stereoscopic microscope. The time taken for male and female *C. noackae* to find each other (s), the copula duration (s) and the number of copulas were recorded.

Oviposition behaviour

Newly emerged *C. noackae* virgin or mated females (< 3 hours old), were individualized in a transparent polystyrene vial (7.5 cm high x 3.0 cm in diameter) with a filter paper strip moistened with 50% honey solution as food. Ten *T. peregrinus* eggs, oviposited on paper towel strips and up to 24 hours old, were numbered from 1 to 10 with a pencil (to facilitate visualization and observation, under a stereoscopic microscope, of the egg handled by the female parasitoid) and offered to each *C. noackae* female for 1 hour at a temperature of 24 ± 2 °C and RH of 60 ± 10%. The eggs in which the parasitoid inserted the ovipositor within 1 hour of evaluation were transferred to individual transparent polystyrene vials (3.5 cm high x 2.0 cm in diameter) and stored at 24 ± 2 ºC, RH of 60 ± 10% and with a photophase of 12 h, until the emergence of *C. noackae* adults or *T. peregrinus* nymphs.

The oviposition behaviour of virgin or mated *C. noackae* females was observed for 1 hour per female under a stereoscopic microscope, timing the period taken to (a) localize (contact) the first host (egg); (b) localize the other hosts (eggs); (c) perform an external evaluation of the host (egg) (females walking on eggs touching them with antennas and tarsomeres); (d) insert the ovipositor in the host. The proportion of eggs with ovipositor insertion (%), parasitism (%), viability (%), sex ratio and frequency (%) of ovipositor insertion by *C. noackae* per *T. peregrinus* egg site were also evaluated.

The proportion of *T. peregrinus* eggs with ovipositor insertion by *C. noackae* (Ins), parasitism (P), viability (V) and sex ratio (rs) were calculated with the following formulas: Ins= [(number of eggs inserted/number of eggs offered)] * 100; P= [(number of emerged parasitoids + retained parasitoids/number of eggs offered)] * 100; V= [(number of parasitoids emerged/number of parasitized eggs)] * 100; rs= number of females/(number of females + number of males), respectively. *Thaumastocoris peregrinus* eggs were dissected after 30 days to obtain the number of parasitoids unmerged therein.

The experimental design was completely randomized with two treatments (virgin or mated females) and 10 replicates (each with 10 *T. peregrinus* eggs: 1 female of *C. noackae*).

Statistical analysis

Before the analysis, data were tested for normality and homoscedasticity of variances. The statistical analysis was performed using an unpaired t-test (*p*-value < 0.05 was considered significant) and the Mann-Whitney U test was used as the nonparametric version of the t-test since the datasets were not normally distributed. A two-way ANOVA was conducted to compare the effects of mating of *C. noackae* females and oviposition sites

and the interaction effect between the mating of *C. noackae* females and oviposition sites on frequency of the ovipositor insertion sites on *T. peregrinus* eggs. Data was processed and analysed using Sigma Plot v. 11.0. for Windows software.

Results

Courtship and mating behaviour

Cleruchoides noackae males and females mated soon after emergence when placed in polystyrene vials, but without defined courtship behaviour. The duration of male-female encounters ranged from 39 to 1.140 seconds (mean of 341.65 seconds) in these bottles. The male touched the female with the antennae and, in seconds, assumed the copula position, mounting and inserting the aedeagus in the reproductive tract of the female, which remained, during copulation, in a walking position, without moving. The male, after mating and during copula, was in the opposite direction to that of the female, with the ventral part of the abdomen upwards, leaning the wings on the base of the polystyrene bottle. Each *C. noackae* couple had only one copula in 1 hour. The *C. noackae* copula lasted 39 seconds, ranging from 29 to 50 seconds. After copulation, males and females separated and walked in the vial.

Oviposition behaviour

Virgin and mated females found the first host egg in a similar delay ($t(18) = -0.326$, $p = 0.748$, Table 1), but mated ones took longer to find the other host eggs (Mann-Whitney U= 14.000, d.f.= 1.18, $p = 0.013$, Table 1). *Cleruchoides noackae* females circled and repeatedly touched *T. peregrinus* eggs with antennae before oviposition for a similar

period between 18 to 31 and 15 to 34 seconds for virgins and mated females (t(18)= 1.326, *p*= 0.202, Table 1), respectively.

Table 1. Time period (mean ± standard error) of localization of the first egg, other eggs, insertion of ovipositor in minutes, and evaluation of the egg in seconds per *Cleruchoides noackae* (Hymenoptera: Mymaridae) female on *Thaumastocoris peregrinus* (Hemiptera: Thaumastocoridae) eggs

Female	First egg (min)[a]	Other eggs (min)[b]	Evaluation (s)[a]	Insertion (min)[b]
Virgin	15.21 ± 4.79[NS]	3.85 ± 2.20[*]	24.49 ± 1.56[NS]	5.13 ± 1.09[NS]
Mated	17.14 ± 3.69	0.86 ± 0.05	21.20 ± 1.85	3.69 ± 0.31

Significant differences as determined by the unpaired t-test ([a]) or Mann-Whitney U test ([b]) are indicated with * ($p<0.05$) and non-significant differences with NS.

Cleruchoides noackae females inserted the ovipositor into the *T. peregrinus* egg immediately after evaluating it. This parasitoid, during oviposition, kept the antennae parallel to the abdomen and the wings unmoved and made vertical abdominal movements lowering and raising the abdomen, characterizing oviposition. The time between ovipositor insertion and withdrawal by virgin or mated *C. noackae* females ranged from 2.34 to 11.95 and from 2.67 to 5.96 minutes, respectively (Mann-Whitney U= 34.000, d.f.= 1.18, *p*= 0.391, Table 1).

Most *C. noackae* females oviposited once per *T. peregrinus* egg, but some oviposited on the same egg (superparasitism characteristics), and one mated female did not locate eggs within 1 hour. Virgin or mated *C. noackae* females inserted the ovipositor in more than 50% of the *T. peregrinus* eggs offered (t(18)= -0.411, *p*= 0.686, Table 2) and

most of them inserted eggs and effectively parasitized during the evaluation period (1 hour/female) (t(18)= -0.596, *p*= 0.576, Table 2).

Table 2. Eggs inserted (%), parasitism (%), viability (%) and sex ratio (mean ± standard error) of virgin and mated *Cleruchoides noackae* (Hymenoptera: Mymaridae) females on *Thaumastocoris peregrinus* (Hemiptera: Thaumastocoridae) eggs during one hour of evaluation

Female	Eggs inserted (%)[a]	Parasitism (%)[a]	Viability (%)[b]	Sex ratio[b]
Virgin	59.00 ± 7.66 NS	55.00 ± 7.78 NS	97.13 ± 1.89 NS	0.00 ± 0.00*
Mated	64.00 ± 9.45	62.00 ± 9.52	75.98 ± 12.12	0.68 ± 0.02

Significant differences as determined by the unpaired t-test ([a]) or Mann-Whitney U test ([b]) are indicated with * ($p<0.05$) and not significant differences with NS.

One *C. noackae* adult emerged per egg, and the viability of *T. peregrinus* eggs parasitized by virgin and mated *C. noackae* was similar (Mann-Whitney U= 46.500, d.f.= 1.18, *p*= 0.790, Table 2). Virgin females of *C. noackae* gave origin only to males and mated females produced both males and females with a higher number of females (Mann-Whitney U= 0.000, d.f.= 1.18, *p*< 0.001, Table 2).

The mating of *C. noackae* females and oviposition sites had no significant interactions (two-way ANOVA; F= 2.777, d.f.= 2.56, *p*= 0.072, Table 3). Mating has no impact on the oviposition site preferences for *C. noackae* females (two-way ANOVA; F= 0.000, d.f.= 1.56, *p*= 1.000, Table 3), but the oviposition site between them differed (two-way ANOVA; F= 50.891, d.f.= 2.56, *p*<0.001, Table 3). Virgin females preferred to insert the ovipositor on the sides of the *T. peregrinus* egg, followed by the operculum and

opposite the operculum (F= 28.133, d.f.= 2.29, $P< 0.001$, Table 3). Mated females preferred to insert the ovipositor on the sides and operculum of the egg than the opposite side of the operculum (F= 25.232, d.f.= 2.26, $p<0.0001$, Table 3).

Table 3. Frequency (mean ± standard error) of the ovipositor insertion site per virgin or mated female of the parasitoid *Cleruchoides noackae* (Hymenoptera: Mymaridae) on *Thaumastocoris peregrinus* (Hemiptera: Thaumastocoridae) eggs

Female	Ovipositor insertion site (%)		
	Opposite the operculum	Operculum	Side
Virgin	10.78 ± 2.16 Ac	33.66 ± 4.30 Ab	55.56 ± 4.22 Aa
Mated	3.43 ± 4.13 Ab	46.34 ± 6.02 Aa	50.23 ± 5.57 Aa

Means followed by the same lowercase letter, per line or upper case, per column, do not differ by the two-way ANOVA (Tukey test, $p < 0.05$).

Discussion

Cleruchoides noackae needs to mate shortly after emergence to produce female progeny, due to its capacity of performing arrhenotokous parthenogenesis [24] with production of only males originated from unfertilized eggs [32,33]. This behaviour is expected for pro-ovigenic parasitoids, such as *C. noackae*, due to their reduced longevity [25]. The emergence of *C. noackae* males and females at the same time [34] and the fact that *T. peregrinus* lay their eggs in groups on eucalyptus leaves [35] increase the chances of this parasitoid meeting and mating. The poorly defined courtship behaviour of *C. noackae* is consistent with that of other parasitoids such as *Trichogramma dendrolimi* Matsumura and *Trichogramma papilionis* Nagarkatti (Hymenoptera: Trichogrammatidae) [36] and *Anagrus* spp. (Hymenoptera: Mymaridae), which males mate with the first female due to a

specific pheromone [37]. Females of *C. noackae* [24] and those of *Anagrus breviphragma* Sokya (Hymenoptera: Mymaridae) [38] were not receptive to a second copula, which may be due to the transfer of chemical substances from their seminal fluid and spermatozoa to the female [39,40].

The reduced time taken for *C. noackae* females to find other host eggs after parasitizing the first one is due to the process known as associative learning [26]. Egg parasitoids developed strategies to find the host and parasitize it, such as the detection of chemicals (semiochemicals) associated with the host or the plant damaged [41,42,43]. Associative learning may be related to the perception of chemical (semiochemical) and/or physical (visual or mechanical) stimuli of the first egg parasitized [44,45] and the parasitoid ability to locate, recognize and accept [46, 29, 26, 43] or reject other hosts [47]. Due to the small size of their hosts, female egg parasitoids have developed a capacity to respond to these stimuli to decrease recognition time and increase parasitism capacity in a shorter time [43]. This behaviour pattern was reported for *Anagrus pseudococci* Girault (Hymenoptera: Encyrtidae), which, after gaining experience, was more efficient in seeking hosts by moving faster and taking shorter time to handle the other hosts [48]. *Anaphes iole* Girault (Hymenoptera: Mymaridae) females, with previous oviposition experience were faster to parasitize *Lygus hesperus* Knight (Hemiptera: Lygaeidae) eggs than those without experience [47].

The fact that *C. noackae* females circled and touched the *T. peregrinus* eggs repeatedly with their antennae shows that this parasitoid needs morphological, chemical or sensory stimulation associated with its host to start oviposition [49]. External investigation of the egg by virgin or mated *C. noackae* females, a process known as evaluation [26],

allows the recognition of characteristics such as form, texture or movement of the host to verify its suitability for oviposition or to identify non-volatile chemicals released by other females during previous oviposition, thus avoiding superparasitism [50,47]. The time spent by *C. noackae* groping *T. peregrinus* with its antennae before inserting the ovipositor was similar to that reported for mated females of this parasitoid, taking 35 seconds with this behavior [51]. However, the external evaluation time taken for the female to recognize and parasitize a host may decrease with successive egg handling experiences [52] and the host age due to internal physical and chemical changes in the eggs [53,26] and with the recognition of host chemicals or lesions produced during ovipositor insertion [47]. The mean evaluation time of *Spartocera dentiventris* Berg (Hemiptera: Coreidae) eggs by the parasitoid *Gryon gallardoi* Brèthes (Hymenoptera: Scelionidae) increased with host age, from 78 to 201 seconds in 2 and 12-day-old eggs, respectively [53]. The previous parasitism experience of *A. iole* females decreased the evaluation time of subsequent hosts [47].

The oviposition of *C. noackae* immediately after contact with *T. peregrinus* eggs agrees with a report on this parasitoid, with an ovipositor insertion time of 2 to 10 minutes at 22 ºC for mated females [51]. The temperature and humidity may have affected the time taken for *C. noackae* to perforate the *T. peregrinus* egg chorion and oviposited, since it affects host conditions and modify the parasitoid female behaviour [54,33], as reported for *Anaphes nitens* Girault and *Anaphes inexpectatus* Huber & Prinsloo (Hymenoptera: Mymaridae) on eggs of *Gonipterus platensis* Marelli (Coleoptera: Curculionidae), a eucalyptus exotic pest. The mating, groaning and oviposition of these parasitoids were more frequent between 10 and 25 ºC than at 5 ºC [55]. However, this varies among

parasitoids, since *A. delicatus* inserted the ovipositor in *Prokelisia marginata* Van Duzee (Homoptera: Delphacidae) eggs for 119 seconds (1.98 minutes) [37], a shorter time than that of *C. noackae* on bronze bug eggs. Variations in ovipositor insertion time, among parasitoid species, may be due to differences in host egg thickness, which affects cuticle perforation time [56].

Virgin or mated *C. noackae* females typically performed only one oviposition per *T. peregrinus* egg and rarely returned to the same egg; this behaviour was also observed when 20 *T. peregrinus* eggs were offered to females of this parasitoid in a glass vial (7.5 cm long x 2.5 cm wide) [24], while the frequency of return to and oviposition in the same egg was higher when 5 eggs were offered [51]. This behaviour may be associated with the need of the parasitoid to increase its fitness and reproductive success [57], and many marked host eggs with chemical substances before releasing them [58,59]. This allows other females to recognize internal chemical changes of parasitized eggs [26], reducing the chances of superparasitism [60]. The non-recognition of co-specific markers and, consequently, the return of female parasitoids to previously oviposited eggs may be due to pheromone degradation and low experience of the female related to oviposition [61,62].

Cleruchoides noackae oviposition soon after emergence is a common pattern for pro-ovigenic parasitoids [34], allowing them to produce offspring more quickly. However, parasitoids may exhibit time and/or egg deposition limitations on their hosts, which force them to develop strategies for host evaluation and oviposition to increase their fecundity and thus, their fitness [49].

The emergence of one *C. noackae* adult per *T. peregrinus* egg is a common pattern for this species; however, this may vary with host volume, species and quality and

may influence parasitoid size [63,64]. The emergence of only one *C. noackae* individual per *T. peregrinus* egg can be explained by the fact that eggs of this pest are 0.48 mm long [65] and the *C. noackae*, 0.5 mm (after emerging) [22].

A sex ratio of 0.68 (in the progeny of mated females) is interesting because parasitoid females are responsible for parasitism, but the reproduction of *C. noackae* by arrhenotokous parthenogenesis is common among Mymaridae species [34]. However, females of the Hymenoptera order can adjust the sex ratio according to host size, age, quality, competition, temperature and oviposition sequence [66,67,68], to increase their reproductive success.

The preference of *C. noackae* for insertion sites on the sides and operculum of *T. peregrinus* eggs is not due to the chorion thickness, which is approximately 0.44 mm all over the egg surface [65]. However, the operculum presents circular projections, probably aeromichromatic and smooth in texture in the outermost region of the egg, which may facilitate the penetration of the *C. noackae* ovipositor into the *T. peregrinus* egg [65]. In addition, this preference may be due to the fact that the *T. peregrinus* egg air layer has irregular columns from the endochorion up to the chorionic layer and the position of the *T. peregrinus* embryo inside the egg where the veal and wax coat is larger in the dorsal region of the egg than in the anterior (operculum) and lateral [65], which facilitates contact of the parasitoid ovipositor with the pest embryo.

Conclusions

Male and female *C. noackae* did not exhibit defined courtship behaviour and mated in less than 1 hour after making initial contact. The time taken to locate the first host,

perform an external evaluation and insert the ovipositor (oviposition), as well as the percentage of inserted, parasitized and viable eggs were similar for virgin and mated *C. noackae* females. The sex ratio and the localization time of the other hosts were higher for mated and virgin females, respectively. The results obtained contribute to improving strategies of *C. noackae* rearing and release in biological control programs for *T. peregrinus*.

Acknowledgements

To Proof-Reading-Services.com for revising and correcting the English language used in this manuscript.

References

1. Wingfield MJ, Slippers B, Hurley BP, Coutinho TA, Wingfield BD, Roux J. Eucalypt pests and diseases: growing threats to plantation productivity. South For. 2008; 70(2): 139-144.
2. Fernandes BF, Barcelos JAV, Andrade HB, Zanuncio JC. *Leptocybe invasa* (Hymenoptera: Eulophidae), an exotic pest of eucalyptus, in Minas Gerais State, Brazil. Fla Entomol. 2014; 97(2): 824-826.
3. Wilcken CF, Barbosa LR, Velozo SM, Becchi LK, Junqueira LR, de Sá LAN, et al. Biological control of *Thaumastocoris peregrinus* (Hemiptera: Thaumastocoridae) in eucalyptus plantations in Brazil: An Update. In: Mason PG, Gillespie DR, Vicent C,

editors. Proceedings of the 5th International Symposium on Biological Control of Arthropods; 2017 Sept 11-15; Langkawi, Malaysia: CABI Wallingford UK, 2017; 6:105-107.

4. Wilcken CF, Soliman EP, de Sá LAN, Barbosa LR, Dias TKR, Ferreira Filho PJ, et al. Bronze bug *Thaumastocoris peregrinus* Carpintero and Dellapé (Hemiptera: Thaumastocoridae) on eucalyptus in Brazil and its distribution. J Plant Prot Res. 2010; 50(2): 201-205.
5. Almeida KECD, Silva JGSD, Silva IMDA, Costa ALD, Laia MLD. Ecophysiological analysis of *Eucalyptus camaldulensis* (Dehnh) submitted to attack from *Thaumastocoris peregrinus* (Carpintero & Dellape). Rev Árvore. 2018; 42(1): e420120.
6. Barbosa LR, Rodrigues AP, Souza LN, Foester LA, Souza AR, Castro BMC, et al. Development of *Cleruchoides noackae*, an egg-parasitoid of *Thaumastocoris peregrinus*, in eggs laid on different substrates, with different ages and post-cold storage. BioControl 2018; 63(2): 193-202.
7. Carpintero DL, Dellapé PM. A new species of *Thaumastocoris* Kirkaldy from Argentina (Heteroptera: Thaumastocoridae: Thaumastocorinae). Zootaxa 2006; 1228(1):61-68.
8. Noack A, Rose H. Life-history of *Thaumastocoris peregrinus* and *Thaumastocoris* sp. in the laboratory with some observations on behaviour. Gen App Entomol. 2007; 36:27-33.

9. Button G. *Thaumastocoris peregrinus*. Forestry facts [online]. Pietermaritzburg: NCT Forestry Co-operative Limited. Apr 2007; 63:16-17. Available from: http://www.nctforest.com/upload/Publications/NV%202007%20April.pdf.
10. Soliman EP, Wilcken CF, Pereira JM, Dias TK, Zaché B, Dal Pogetto MH, et al. Biology of *Thaumastocoris peregrinus* in different eucalyptus species and hybrids. Phytoparasitica 2012; 40(3): 223-230.
11. Lima ACV, Wilcken CF, Ferreira-Filho PJ, Serrão JE, Zanuncio JC. Intra-plant spatial distribution of *Thaumastocoris peregrinus* Carpintero & Dellapé (Hemiptera: Thaumastocoridae) on *Eucalyptus grandis* plants. Phytoparasitica 2016; 44(3): 411-418.
12. Wilcken CF, Barbosa LR, Soliman EP, Lima ACV, de Sá LAN, Lawson S. Percevejo-bronzeado-do-eucalipto, *Thaumastocoris peregrinus* Carpinteiro & Dellapé. In: Vilela EF, Zucchi RA, editors. Pragas Introduzidas No Brasil: Insetos e Ácaros, FEALQ; 2015. pp. 898-908.
13. Zanuncio JC, do Nascimento EC, Garcia JF. Major lepidopterous defoliators of eucalyptus, in the Southeast Brasil. For Ecol Manage. 1994; 65(1): 53-63.
14. Nadel RL, Noack AE. Current understanding of the biology of *Thaumastocoris peregrinus* in the quest for a management strategy. Int J Pest Manag. 2012; 58(3): 257-266.
15. Mascarin GM, Duarte VS, Brandão MM, Delalíbera JrI. Natural occurrence of *Zoophthora radicans* (Entomophthorales: Entomophthoraceae) on *Thaumastocoris peregrinus* (Heteroptera: Thaumastocoridae), an invasive pest recently found in Brazil. J Invertebr Pathol. 2012; 110(3): 401–404.

16. Lorencetti GAT, Potrich M, Mazaro SM, Lozano ER, Barbosa LR, Menezes MJS, et al. Eficiência de *Beauveria bassiana* Vuill. e *Isaria* sp. para o controle de *Thaumastocoris peregrinus* Carpintero & Dellapé (Hemiptera: Thaumastocoridae). Ciênc Florest. 2018; 28(1): 403-411.
17. Soliman EP, Castro BMC, Wilcken CF, Firmino AC, Pogetto MHFAD, Barbosa LR, Zanuncio JC. Susceptibility of *Thaumastocoris peregrinus* (Hemiptera: Thaumastocoridae), a eucalyptus pest, to entomopathogenic fungi. Sci Agric. 2019; 76(3): 255-260.
18. Barbosa LR, Santos F, Barddal HPO, Machado BO, Wilcken CF, Soliman EP. Predação de *Thaumastocoris peregrinus* por *Chrysoperla externa*. Comunicado técnico Embrapa Florestas. Nov 2010; 257(1): 1-4. Available from: https://www.infoteca.cnptia.embrapa.br/bitstream/doc/870870/1/CT257.pdf.
19. Souza GK, Pikart TG, Pikart FC, Serrão JE, Wilcken CF, Zanuncio JC. First record of a native heteropteran preying on the introduced eucalyptus pest, *Thaumastocoris peregrinus* (Hemiptera: Thaumastocoridae), in Brazil. Fla Entomol. 2012; 95(2): 517-520.
20. Dias TKR, Wilcken CF, Soliman EE, Barbosa LR, Serrão JE, Zanuncio JC. Predation of *Thaumastocoris peregrinus* (Hemiptera: Thaumastocoridae) by *Atopozelus opsimus* (Hemiptera: Reduviidae) in Brazil. Invertebrate Surviv J. 2014; 11(1): 224-227.
21. Cross D. The parasitoids of *Thaumastocoris* spp. in the Sydney region. M.Sc. Thesis, Department of Food and Natural Resources: University of Sydney, Australia. 2009.

22. Lin NQ, Huber JT, Salle JL. The australian genera of Mymaridae (Hymenoptera: Chalcidoidae). Zootaxa 2007; 1596(1): 1-111.

23. Barbosa LR, Rodrigues AP, Soler LS, Fernandes BV, Castro BMC, Wilcken CF, et al. Establishment in the field of *Cleruchoides noackae* (Hymenoptera: Mymaridae), an exotic egg parasitoid of *Thaumastocoris peregrinus* (Hemiptera: Thaumastocoridae). Fla Entomol. 2017; 100(2): 372-374.

24. Mutitu EK, Garnas JR, Hurley BP, Wingfield MJ, Harney M, Bush SJ, et al. Biology and rearing of *Cleruchoides noackae* (Hymenoptera: Mymaridae), an egg parasitoid for the biological control of *Thaumastocoris peregrinus* (Hemiptera: Thaumastocoridae). J Econ Entomol. 2013; 106(5): 1979-1985.

25. Souza AR, Candelaria MC, Barbosa LR, Wilcken CF, Campos JM, Serrão JE, et al. Longevity of *Cleruchoides noackae* (Hymenoptera: Mymaridae), an egg parasitoid of *Thaumastocoris peregrinus* (Hemiptera: Thaumastocoridae), with various honey concentrations and at several temperatures. Fla Entomol. 2016; 99(1): 33-37.

26. Vinson SB. The general host selection behaviour of parasitoid Hymenoptera and a comparison of initial strategies utilized by larvaphagous and oophagous species. Biol Control 1998; 11(2): 79-96.

27. Beserra E, Parra JRP. Comportamento de parasitismo de *Trichogramma atopovirilia* Oatman & Platner e *Trichogramma pretiosum* Riley (Hymenoptera, Trichogrammatidae) em posturas de *Spodoptera frugiperda* (J.E. Smith) (Lepidoptera, Noctuidae). Rev Bras Entomol. 2003; 47(2): 205-209.

28. Corbet SA. Insect chemosensory responses: a chemical legacy hypothesis. Ecol Entomol. 1985; 10(2): 143-153.

29. Nurindah BWC, Gordh G. Experience acquisition by *Trichogramma australicum* Girault (Hymenoptera: Trichogrammatidae). Aust J Entomol. 1999; 38(2): 115-119.
30. Barbosa LR, Santos F, Buhrer CB, Nichele LA, Wilcken CF, Soliman EP. Criação massal do percevejo bronzeado, *Thaumastocoris peregrinus*: Carpinteiro and Dellapé, 2006 (Hemiptera, Thaumastocoridae). Folheto, Embrapa Florestas. Jun 2016; 22p. Available from: https://ainfo.cnptia.embrapa.br/digital/bitstream/item/145907/1/Criacao-massal-do-percevejo-bronzeado.pdf.
31. Barbosa LR, Rodrigues AP, Nichele LA, Souza AR, Becchi LK, Wilcken CF. Orientações para a criação massal e liberação em campo de *Cleruchoides noackae* para controle biológico do percevejo bronzeado do eucalipto. Folheto, Embrapa Florestas. 2017; 23p. Available from: https://ainfo.cnptia.embrapa.br/digital/bitstream/item/167917/1/Livro-TA-1393-completo.pdf.
32. Rabeling C, Kronauer DJC. Thelytokous parthenogenesis in eusocial Hymenoptera. Annu Rev Entomol. 2013; (58): 273-292.
33. Gullan PJ, Cranston PS. Insetos - Fundamentos da Entomologia. 5th ed. Rio de Janeiro: Roca; 2017.
34. Jervis MA, Heimpel GE, Ferrns PN. Life-history strategies in parasitoid wasps: a comparative analysis of “ovigeny”. J Anim Ecol. 2001; 70(3): 442-458.

35. Jacobs DH, Neser S. *Thaumastocoris australicus* Kirkaldy (Heteroptera: Thaumastocoridae): a new insect arrival in South Africa, damaging to eucalyptus trees: research in action. S Afr J Sci. 2005; 101(5): 233-236.

36. Suzuki Y, Hiehata K. Mating systems and sex ratios in the egg parasitoids, *Trichogramma dendrolimi* and *T. papilionis* (Hymenoptera: Trichogrammatidae). Anim Behav. 1985; 33(4): 1223-1227.

37. Cronin JT, Strong DR. Biology of *Anagrus delicatus* (Hymenoptera: Mymaridae) an egg parasitoid of *Prokelisia marginata*. Ann Entomol Soc Am. 1990; 83(4): 846-854.

38. Usmani MK. Biological investigations on some species of *Anagrus* (Hymenoptera, Mymaridae), egg parasitoids of leafhoppers (Hemiptera). APCBEE Procedia 2012; (4): 1-5.

39. Davey KG. The female reproductive tract. In: Kerkut GA, Gilbert LI, editors. Comprehensive insect physiology, biochemistry and pharmacology. New York: Pergamon Press, Elmsford; 1985. pp. 15-36.

40. Neubaum DM, Wolfner MF. Wise, winsome, or weird? Mechanisms of sperm storage in female animals. Curr Top Dev Biol. 1999; (41): 67-97.

41. Fatouros NE, Dicke M, Mumm R, Meiners T, Hilker M. Foraging behavior of egg parasitoids exploiting chemical information. Behav Ecol. 2008; 19(3): 677–689.

42. Chiappini E, Salerno G, Berzolla A, Iacovone A, Reguzzi MC, Conti E. Role of volatile semiochemicals in host location by the egg parasitoid *Anagrus breviphragma*. Entomol Exp Appl. 2012; 144(3): 311-316.

43. Conti E, Collazza S. Chemical ecology of egg parasitoids associated with true bugs. Psyche J Entomol. 2012; (2012): 1-11.

44. Godfray HCJ. Parasitoids, behavioral and evolutionary ecology. Princeton University Press; 1994. 67: 1-473.

45. Dindo ML, Nakamura S. Oviposition strategies of tachinid parasitoids: two Exorista species as case studies. Int J Insect Sci. 2018; 10: 1-6.

46. Kaiser L, Pham-delegue MH, Masson C. Behavioural study of plasticity in host preferences of *Trichogramma maidis* (Hym.: Trichogrammatidae). Physiol Entomol. 1989; 14(1): 53-60.

47. Conti E, Jones WA, Bin F, Vinson SB. Oviposition behavior of *Anaphes iole*, an egg parasitoid of *Lygus hesperus* (Hymenoptera: Mymaridae; Heteroptera: Miridae). Ann Entomol Soc Am. 1997; 90(1): 91-101.

48. Islam KS. The influence of host and other environmental factors on the biology of the mealybug parasitoid *Anagyrus pseudococci* (Girault). PhD Thesis, University of London, United Kingdom; 1993.

49. Fellowes MDE, van Alphen JJM, Jervis MA. Foraging behavior. In: Jervis MA, editor. Insects as natural enemies. Springer, Dordrecht; 2005. pp. 1-72.

50. Vinson SB. Chemical signals used by parasitoids. Redia 1991; (74): 15-42.

51. Haas J, Barbosa LR, Potrich M, Lozano ER, Mazaro SM. Oviposition behavior of *Cleruchoides noackae* (Hymenoptera: Mymaridae) in the laboratory. Floresta e Ambiente 2018; 25(1): e00148115.

52. Wajnberg E. Analysis of variations of handling-time in *Trichogramma maidis*. Entomophaga 1989; 34(3): 397-407.

53. Rocha L, Redaelli L, Mendonça Jr MS. Oviposition behaviour of *Gryon gallardoi* (Hymenoptera, Scelionidae) on eggs of different ages of *Spartocera dentiventris* (Hemiptera, Coreidae). Iheringia Ser Zool. 2006; 96(3): 277-282.
54. Waage JK. Sib-mating and sex ratio strategies in scelionid wasps. Ecol Entomol. 1982; 7(1): 103-112.
55. Valente C, Gonçalves CI, Reis A, Branco M. Pre-selection and biological potential of the egg parasitoid *Anaphes inexpectatus* for the control of the eucalyptus snout beetle, *Gonipterus platensis*. J Pest Sci. 2017; 90(3): 911-923.
56. Vinson SB. The behavior of parasitoids. In: Kerkut GA, Gilbert LI, editors. Comprehensive insect physiology, biochemistry and pharmacology. New York: Pergamon Press, Elmsford; 1985. pp. 417-469.
57. Moreira MD, Santos MCF, Beserra EB, Torres JB, Almeida RP. Parasitismo e superparasitismo de *Trichogramma pretiosum* Riley (Hymenoptera: Trichogrammatidae) em ovos de *Sitotroga cerealella* (Oliver) (Lepidoptera: Gelechiidae). Neotrop Entomol. 2009; 38(2): 237-242.
58. Strand MR, Ratner S, Vinson SB. Maternally induced host regulation by the egg parasitoid *Telenomus heliothidis*. Physiol Entomol. 1980; 8(4): 469-475.
59. Tanaka T. Calyx and venom fluids of *Apanteles kariyai* (Hymenoptera: Braconidae) as factors that prolong larval period of the host, *Pseudaletis separata* (Lepidoptera: Noctuidae). Ann Entomol Soc Am. 1987; 80(4): 530-533.
60. Darrouzet E, Bignon L, Chevrier C. Impact of mating status on egg-laying and superparasitism behaviour in a parasitoid wasp. Entomol Exp Appl. 2007; 123(3): 279-285.

61. Salt G. Experimental studies in insect parasitism. II. Superparasitism. P Roy Soc Lond B. 1934; 114(790): 455-476.

62. Okuda MS, Yeargan KV. Intra-and interspecific host discrimination in *Telenomus podisi* and *Trissolcus euschisti* (Hymenoptera: Scelionidae). Ann Entomol Soc Am. 1988; 81(6): 1017-1020.

63. Vinson SB. Comportamento de seleção hospedeira de parasitoides de ovos, com ênfase na família Trichogrammatidae. In: Parra JRP, Zucchi RA, editors. *Trichogramma* e o controle biológico aplicado. Piracicaba, FEALQ. 1997; pp. 67-119.

64. Bueno RCOF, Parra JRP, Bueno AF, Haddad M. Desempenho de tricogramatídeos como potenciais agentes de controle de *Pseudoplusia includens* Walker (Lepidoptera: Noctuidae). Neotrop Entomol. 2009; 38(3): 389-394.

65. Souza GK. Morfologia de ovos, glândulas salivares e sistemas digestivo e reprodutor de *Thaumastocoris peregrinus* (Hemiptera: Thaumastocoridae). M.Sc. Thesis. Entomology department: Federal University of Viçosa, Viçosa, Brazil; 2012.

66. Waage JK. Family planning in parasitoids: Adaptive patterns of progeny and sex allocation. In: Waage J, Greatheat D, editors. Insect Parasitoids. Academic Press; 1986. pp. 63-96.

67. King BH. Offspring sex ratios in parasitoid wasps. Q Rev Biol. 1987; 62(4): 367-396.

68. Van Baaren J, Nénon JP, Boivin G. Comparison of oviposition behavior of a solitary and a gregarious parasitoid (Hymenoptera: Mymaridae). J Insect Behav. 1995; 8(5): 671-686.

Supporting information

S1_Table. Time period (mean ± standard error) of localization of the first egg, other eggs, insertion of ovipositor in minutes, and evaluation of the egg in seconds per *Cleruchoides noackae* (Hymenoptera: Mymaridae) female on *Thaumastocoris peregrinus* (Hemiptera: Thaumastocoridae) eggs. Significant differences as determined by the unpaired t-test (a) or Mann-Whitney U test (b) are indicated with * ($p<0.05$) and non-significant differences with NS.

S2_Table. Eggs inserted (%), parasitism (%), viability (%) and sex ratio (mean ± standard error) of virgin and mated *Cleruchoides noackae* (Hymenoptera: Mymaridae) females on *Thaumastocoris peregrinus* (Hemiptera: Thaumastocoridae) eggs during one hour of evaluation. Significant differences as determined by the unpaired t-test (a) or Mann-Whitney U test (b) are indicated with * ($p<0.05$) and not significant differences with NS.

S3_Table. Frequency (mean ± standard error) of the ovipositor insertion site per virgin or mated female of the parasitoid *Cleruchoides noackae* (Hymenoptera: Mymaridae) on *Thaumastocoris peregrinus* (Hemiptera: Thaumastocoridae) eggs.

Means followed by the same lowercase letter, per line or upper case, per column, do not differ by the two-way ANOVA (Tukey test, $p < 0.05$).

S1_Dataset_Table. Dataset of time period of localization, insertion of ovipositor and evaluation of the *T. peregrinus* eggs per *Cleruchoides noackae* female.

S2_Dataset_Table. Dataset of eggs inserted, parasitism, viability and sex ratio of virgin and mated *C. noackae.*

S3_Dataset_Table. Dataset of frequency of the ovipositor insertion site per virgin or mated female of the parasitoid *C. noackae.*

Supporting Information Table 3

Click here to access/download
Supporting Information
S3_Table..xlsx

Supporting Information Table 1

Click here to access/download
Supporting Information
S1_Table..xlsx

Supporting Information Table 2

Click here to access/download
Supporting Information
S2_Table..xlsx

Dataset_Tabel3

Click here to access/download
Supporting Information
S3_Dataset_Table.3.xlsx

Dataset_Table1

Click here to access/download
Supporting Information
S1_Dataset_Table.1.xlsx

Dataset_Table2

Click here to access/download
Supporting Information
S2_Dataset_Table.2.xlsx
