# Supplementary material for: Oviposition behaviour of mated or unmated Cleruchoides noackae (Hymenoptera: Mymaridae)
Source: PLoS One. 2020 Oct 2;15(10):e0239285. doi: 10.1371/journal.pone.0239285 (PMC7531830; doi:10.1371/journal.pone.0239285)
Supplement: Attachment — Submitted filename: Oviposition behaviour of C. noackae_Review.docx [file pone.0239285.s004.docx]

**Oviposition behaviour of mated or unmated *Cleruchoides noackae* (Hymenoptera: Mymaridae) females**

Luciane Katarine Becchi^1¶*^, Carolina Jorge^2¶^, Gabriella Ferreira de Camargo^3&^, Leonardo Rodrigues Barbosa^4¶^, Marcus Alvarenga Soares^5&^, José Eduardo Serrão^6&^, José Cola Zanuncio^7¶^, Carlos Frederico Wilcken^1¶^

^1^Departamento de Proteção Vegetal, Faculdade de Ciências Agronômicas, Universidade Estadual Paulista (UNESP), Campus de Botucatu, Botucatu, São Paulo, Brasil.

^2^Instituto Superior de Estudios Forestales. CENUR Noreste Sede Tacuarembó, Universidad de la República, Tacuarembó, Uruguay.

^3^Suzano Papel e Celulose - Unidade Mucuri. Mucuri, Bahia, Brasil.

^4^Empresa Brasileira de Pesquisa Agropecuária–Embrapa Florestas, Colombo, Paraná, Brasil.

^5^Programa de Pós-Graduação em Produção Vegetal, Universidade Federal dos Vales Jequitinhonha e Mucuri (UFVJM), Diamantina, Minas Gerais, Brasil.

^6^Departamento de Biologia Geral/BIOAGRO, Universidade Federal de Viçosa, Viçosa, Minas Gerais, Brasil.

^7^Departamento de Entomologia/BIOAGRO, Universidade Federal de Viçosa, Viçosa, Minas Gerais, Brasil.

* Corresponding author

E-mail: [becchiluciane@](mailto:becchiluciane@)gmail.com (LKB)

^¶^These authors contributed equally to this work.

^&^These authors also contributed equally to this work.

**Abstract**

*Cleruchoides noackae* (Hymenoptera: Mymaridae), native to Australia, is the most promising biological control agent for *Thaumastocoris peregrinus* (Hemiptera: Thaumastocoridae), an exotic *Eucalyptus* spp. pest. The objective of this research was to study the courtship behaviour, mating and oviposition of virgin or mated *C. noackae* females parasitizing *T. peregrinus* eggs. Virgin or mated females were placed individually in polystyrene flasks with 10 *T. peregrinus* eggs each, and the oviposition behaviour, percentage of eggs inserted and parasitized, viability and sex ratio of emerged *C. noackae* were recorded. This species lacked defined courtship behaviour and mated in less than an hour after emergence. The time spent finding the first host, evaluating the host and inserting the ovipositor and the percentage of inserted and parasitized eggs and their viability were similar for mated and unmated *C. noackae* females. The time taken to locate other host eggs and the sex ratio (female: male) of the offspring were higher for mated females. The ability of *C. noackae* to mate and oviposit in less than 1 hour and the parasitism of *T. peregrinus* eggs by virgin and mated females can improve breeding and biological control of *T. peregrinus*.

**Introduction**

Australian exotic pest insects damage forest plantations in South America, especially those of the genus *Eucalyptus* (Myrtaceae) [1,2,3]. The bronze bug, *Thaumastocoris peregrinus* Carpintero & Dellapé, 2006 (Hemiptera: Thaumastocoridae), detected in Brazil in 2008 in the states of São Paulo and Rio Grande do Sul, has since spread rapidly throughout the country [4,5,6]. This exotic insect pest of *Eucalyptus* has a gregarious and sucking habit and damages eucalyptus trees [7,8]. Symptoms caused by this pest include silvering, tanning and drying of leaves due to sap sucking, followed by defoliation of susceptible genotypes [9,4,10]. In addition, reduction in tree diameter, height and volume, besides losses in eucalyptus wood yield of up to 10%-15% were recorded in eucalyptus plantations infested by *T. peregrinus* [4, 3].

*Thaumastocoris peregrinus* is monitored by sampling the leaves from the middle third on the east side of the eucalyptus trees, counting nymphs, adults and/or eggs of this pest [11] or by using yellow sticky traps [12].

Biological control is the most suitable strategy for *T. peregrinus* management in eucalyptus plantations [12], due to sustainability certification requirements for planted areas, lower environmental risks and the high cost of chemical products [13, 14]. Entomopathogenic fungi cause high *T. peregrinus* nymph and adult mortality in the laboratory and also in epizootics in the field [15,16,17]. Native predatory insects such as *Chrysoperla externa* Hagen (Neuroptera: Chrysopidae) [18], *Supputius cincticeps* Stal (Heteroptera: Pentatomidae) [19] and *Atopozelus opsimus* Elkins (Hemiptera: Reduviidae) [20] prey on nymphs and adults of *T. peregrinus* in Brazil. The egg parasitoid, *Cleruchoides noackae* Lin & Huber, 2007 (Hymenoptera: Mymaridae), the main biological control agent of the bronze bug [21,22,14], was introduced to Brazil in 2012 for classical biological control of this pest [6].

*Cleruchoides noackae* is a solitary endoparasitoid, approximately 0.5 mm long [22], and parasitizes *T. peregrinus* eggs from zero to three days old with an emergence percentage above 60% [6]. The parasitism of *T. peregrinus* by *C. noackae* in the laboratory and the field is 50%-60% [23] and its release in eucalyptus plantations in Brazil reduced the infestation by this pest [3].

*Cleruchoides noackae* reproduce by arrhenotokous parthenogenesis, with fertilized eggs giving females and unfertilized eggs giving males [24]. In the field, the sex ratio (female: male) of this parasitoid, emerging from *T. peregrinus* eggs, was 0.65 [23]. *Cleruchoides noackae* reared in the laboratory has a short longevity, 1.1 to 3.6 days without and with food, respectively [25]. The reproductive behaviour of *C. noackae* needs further studies, and it is not known when males mate with females in flasks that have been used in the laboratory to rear this parasitoid.

Females of egg parasitoids exhibit distinct oviposition behaviour, consisting of host location and evaluation, ovipositor insertion, host acceptance, oviposition and chemical or mechanical marking to avoid superparasitism [26]. These patterns may be inborn to the species or learned by experience during the host evaluation and oviposition process [27]. The female can, by learning, locate and parasitize the host more efficiently and quickly [28,29]. Therefore, the mating behaviour and oviposition patterns of *C. noackae* should be studied for the purposes of efficiently cultivating this parasitoid in the laboratory and increasing its efficacy in the field.

The objective was to evaluate, in the laboratory, the courtship, mating and oviposition behaviour of virgin or mated *C. noackae* females on *T. peregrinus* eggs.

**Materials and Methods**

**Study site**

The bioassays were carried out at the Laboratory of Biological Control of Forest Pests (LCBPF) of the São Paulo State University "Júlio de Mesquita Filho" (UNESP), School of Agricultural Sciences (FCA), Botucatu Campus in Botucatu, São Paulo, Brazil.

**Host *Thaumastocoris peregrinus***

*Thaumastocoris peregrinus* eggs were obtained from LCBPF breeding stocks, which were initiated with eggs, nymphs and adults collected in eucalyptus plantations in the state of Minas Gerais, Brazil. The insects were kept on 3-year-old *Eucalyptus urophylla* var. *platyphylla* (Myrtaceae) branches in 250-ml Erlenmeyer flasks with water on a rectangular plastic tray (40 cm long x 35 cm wide x 8 cm high). Strips of paper towel (1.5 cm wide x 15.0 cm in length) were arranged on the upper surfaces of the branches for 24 hours as a substrate for *T. peregrinus* oviposition. These eggs were used for parasitoid multiplication and experimentation. *Thaumastocoris* *peregrinus* were reared in an air-conditioned room at 24 ± 2 ºC, 60 ± 10% RH and with a 12-h photophase [30].

**Parasitoid *Cleruchoides noackae***

*Cleruchoides noackae* adults, were sourced from LCBPF rearing stock that had been started with *T. peregrinus* parasitized eggs collected in eucalyptus plantations in the state of Rio Grande do Sul, Brazil and were kept in transparent polystyrene bottles (7.5 cm high x 3.0 cm in diameter) with paper filter strips (7.0 cm high x 1.5 cm wide) moistened in 50% honey solution as food. Twenty-four-hour-old *T. peregrinus* eggs were offered to the females of this parasitoid in a climatic chamber at 24 ± 2 ºC, 60 ± 10% RH and with a 12-h photophase [31].

**Courtship and mating behaviour**

One hundred *T. peregrinus* eggs, parasitized by *C. noackae*, were stored individually in transparent polystyrene vials (3.5 cm high x 2.0 cm in diameter) at a temperature of 24 ± 2 ºC; RH of 60 ± 10% and with a photophase of 12 h. The adult parasitoids were sexed shortly after emergence (< 3 hours old), based on the morphology of their antennae: filiform (males) and clavate (female) [22] and received a 50% honey solution. The behaviour, before and during mating, of 11 *C. noackae* virgin couples, placed individually in transparent polystyrene vials (7.5 cm high x 3.0 cm in diameter), was observed for 1 hour under a stereoscopic microscope. The time taken for male and female *C. noackae* to find each other, the copula duration and the number of copulas were recorded.

**Oviposition behaviour**

Newly emerged *C. noackae* virgin or mated females (< 3 hours old), were individualized in a transparent polystyrene vials (7.5 cm high x 3.0 cm in diameter) with a filter paper strip moistened with 50% honey solution as food. Ten *T. peregrinus* eggs, that had been oviposited on paper towel strips and up to 24 hours old, were numbered from 1 to 10 with a pencil (to facilitate visualization and observation, under a stereoscopic microscope, of the egg handled by the female parasitoid) and offered to each *C. noackae* female for 1 hour at a temperature of 24 ± 2 °C and RH of 60 ± 10%. The eggs in which the parasitoid inserted the ovipositor within 1 hour of evaluation were transferred to individual transparent polystyrene vials (3.5 cm high x 2.0 cm in diameter) and stored at 24 ± 2 ºC, RH of 60 ± 10% and with a photophase of 12 h, until the emergence of *C. noackae* adults or *T. peregrinus* nymphs.

The oviposition behaviour of virgin or mated *C. noackae* females was observed for one hour per female under a stereoscopic microscope, timing the period taken to (a) localize (contact) the first host egg; (b) localize the other host eggs; (c) perform an external evaluation of the host egg (females walking on eggs touching them with antennas and tarsomeres); (d) insert the ovipositor in the host. The proportion of eggs with ovipositor insertion (%), parasitism (%), viability (%), sex ratio and frequency (%) of ovipositor insertion by *C. noackae* per *T. peregrinus* egg site were also evaluated.

The proportion of *T. peregrinus* eggs with ovipositor insertion by *C. noackae* (Ins), parasitism (P), viability (V) and sex ratio (rs) were calculated with the following formulas: Ins= [(number of eggs inserted/number of eggs offered)] * 100; P= [(number of emerged parasitoids + retained parasitoids/number of eggs offered)] * 100; V= [(number of parasitoids emerged/number of parasitized eggs)] * 100; rs= number of females/(number of females + number of males), respectively. *Thaumastocoris peregrinus* eggs were dissected after 30 days to obtain the number of parasitoids unmerged therein.

The experimental design was completely randomized with two treatments (virgin or mated females) and 10 replicates (each with 10 *T. peregrinus* eggs: 1 female of *C. noackae*).

**Statistical analysis**

Before the analysis, data were tested for normality and homoscedasticity of variances. The statistical analysis was performed using an unpaired t-test (significant p-value < 0.05) and the Mann-Whitney U test was used as the nonparametric version of the t-test since the datasets were not normally distributed. A two-way ANOVA was conducted to compare the effects of mating of *C. noackae* females and oviposition sites and the interaction effect between the mating of *C. noackae* females and oviposition sites on frequency of the ovipositor insertion sites on *T. peregrinus* eggs. Data was processed and analysed using Sigma Plot v. 11.0. for Windows software.

**Results**

**Courtship and mating behaviour**

*Cleruchoides noackae* males and females mated soon after emergence when placed in polystyrene vials, but without defined courtship behaviour. The duration of male-female encounters ranged from 39 to 1.140 seconds (mean of 341.65 seconds) in these bottles. The male touched the female with its antennae and, in seconds, assumed the copula position, mounting and inserting the aedeagus in the reproductive tract of the female, which remained in a walking position, without moving. The male, after mating and during copula, was aligned in the opposite direction to that of the female, with the ventral part of the abdomen facing upwards, leaning its wings on the base of the polystyrene bottle. Each *C. noackae* couple had only one copula in one hour. Copula in *C. noackae* lasted 39 seconds on average, ranging from 29 to 50 seconds. After copulation, males and females separated and walked about in the vial.

**Oviposition behaviour**

Virgin and mated females found the first host egg with a similar delay (t(18)= -0.326, *p* = 0.748, Table 1), but mated females took longer to find other host eggs (Mann-Whitney U= 14.000, d.f.= 1.18, *p*= 0.013, Table 1). *Cleruchoides noackae* females circled and repeatedly touched *T. peregrinus* eggs with their antennae before ovipositing for a similar period between 18 to 31 and 15 to 34 seconds for virgin and mated females, respectively (t(18)= 1.326, *p*= 0.202, Table 1).

**Table 1. Time period (mean ± standard error) of localization of the first egg, other eggs, insertion of ovipositor in minutes, and evaluation of the egg in seconds per *Cleruchoides noackae* (Hymenoptera: Mymaridae) female on *Thaumastocoris peregrinus* (Hemiptera: Thaumastocoridae) eggs**

| Female | First egg (min)^a^ | Other eggs (min)^b^ | Evaluation (s)^a^ | Insertion (min) ^b^ |
| --- | --- | --- | --- | --- |
| Virgin | 15.21 ± 4.79^NS^ | 3.85 ± 2.20^*^ | 24.49 ± 1.56^NS^ | 5.13 ± 1.09^NS^ |
| Mated | 17.14 ± 3.69 | 0.86 ± 0.05 | 21.20 ± 1.85 | 3.69 ± 0.31 |

Significant differences as determined by the unpaired t-test (^a^) or Mann-Whitney U test (^b^) are indicated with * (*p*<0.05) and non-significant differences with NS.

*Cleruchoides noackae* females inserted their ovipositor into a *T. peregrinus* egg immediately after evaluating it. During oviposition, females kept their antennae parallel to the abdomen with no wing movements and vertically lowered and raised the abdomen, characterizing oviposition. The time between ovipositor insertion and withdrawal by virgin or mated *C. noackae* females ranged from 2.34 to 11.95 and from 2.67 to 5.96 minutes, respectively (Mann-Whitney U= 34.000, d.f.= 1.18, *p*= 0.391, Table 1).

Most *C. noackae* females oviposited once per *T. peregrinus* egg, but some oviposited on the same egg (a superparasitism characteristic), and one mated female did not locate eggs for oviposition within one hour. Virgin or mated *C. noackae* females inserted the ovipositor in more than 50% of the *T. peregrinus* eggs offered (t(18)= -0.411, *p*= 0.686, Table 2) and most of them inserted eggs and effectively parasitized during the evaluation period (1 hour/female) (t(18)= -0.596, *p*= 0.576, Table 2).

**Table 2. Eggs inserted (%), parasitism (%), viability (%) and sex ratio (mean ± standard error) of virgin and mated *Cleruchoides noackae* (Hymenoptera: Mymaridae) females on *Thaumastocoris peregrinus* (Hemiptera: Thaumastocoridae) eggs during one hour of evaluation**

| Female | Eggs inserted (%)^a^ | Parasitism (%)^a^ | Viability (%)^b^ | Sex ratio^b^ |
| --- | --- | --- | --- | --- |
| Virgin | 59.00 ± 7.66 ^NS^ | 55.00 ± 7.78 ^NS^ | 97.13 ± 1.89 ^NS^ | 0.00 ± 0.00* |
| Mated | 64.00 ± 9.45 | 62.00 ± 9.52 | 75.98 ± 12.12 | 0.68 ± 0.02 |

Significant differences as determined by the unpaired t-test (^a^) or Mann-Whitney U test (^b^) are indicated with * (p<0.05) and not significant differences with NS.

One *C. noackae* adult emerged per egg, and the viability of *T. peregrinus* eggs parasitized by virgin and mated *C. noackae* was similar (Mann-Whitney U= 46.500, d.f.= 1.18, *p*= 0.790, Table 2). Virgin females of *C. noackae* only produced males and mated females produced both males and females, with a higher frequency of females (Mann-Whitney U= 0.000, d.f.= 1.18, *p*< 0.001, Table 2).

The mating of *C. noackae* females and oviposition sites had no significant interactions (two-way ANOVA; F= 2.777, d.f.= 2.56, *p=* 0.072, Table 3). Mating had no impact on the oviposition site preferences for *C. noackae* females (two-way ANOVA; F= 0.000, d.f.= 1.56, *p=* 1.000, Table 3), but the oviposition site between them differed (two-way ANOVA; F= 50.891, d.f.= 2.56, *p*<0.001, Table 3). Virgin females preferred to insert the ovipositor on the side of an egg, followed by the operculum and least opposite the operculum (F= 28.133, d.f.= 2.29, *P*< 0.001, Table 3). Mated females preferred to insert the ovipositor equally on the sides and operculum of the egg and least on the opposite side of the operculum (F= 25.232, d.f.= 2.26, *p*<0.0001, Table 3).

**Table 3. Frequency (mean ± standard error) of the ovipositor insertion site per virgin or mated female of the parasitoid *Cleruchoides noackae* (Hymenoptera: Mymaridae) on *Thaumastocoris peregrinus* (Hemiptera: Thaumastocoridae) eggs**

| Female | Ovipositor insertion site (%) | | | |
| --- | --- | --- | --- | --- |
|  | Opposite the operculum | Operculum | Side |  |
| Virgin | 10.78 ± 2.16 Ac | 33.66 ± 4.30 Ab | 55.56 ± 4.22 Aa |  |
| Mated | 3.43 ± 4.13 Ab | 46.34 ± 6.02 Aa | 50.23 ± 5.57 Aa |  |

Means followed by the same lowercase letter, per line or upper case, per column, do not differ by the two-way ANOVA (Tukey test, p < 0.05).

**Discussion**

*Cleruchoides noackae* needs to mate shortly after emergence to produce female progeny, due to its capacity for arrhenotokous parthenogenesis [24] with production of only males originating from unfertilized eggs [32,33]. This behaviour is expected for pro-ovigenic parasitoids, such as *C. noackae*, due to their reduced longevity [25]. The emergence of *C. noackae* males and females at the same time [34] and the fact that *T. peregrinus* lay their eggs in groups on eucalyptus leaves [35] increase the chances of male and female parasitoids meeting and mating. The poorly defined courtship behaviour of *C. noackae* is consistent with that of other parasitoids such as *Trichogramma dendrolimi* Matsumura and *Trichogramma papilionis* Nagarkatti (Hymenoptera: Trichogrammatidae) [36] and *Anagrus* spp. (Hymenoptera: Mymaridae), in which males mate with the first female encountered due to a specific pheromone [37]. Females of *C. noackae* [24] and those of *Anagrus breviphragma* Sokya (Hymenoptera: Mymaridae) [38] were not receptive to a second copula, which may be due to the transfer of chemical substances from male seminal fluid and spermatozoa to the female [39,40].

The reduced time taken for *C. noackae* females to find other host eggs after parasitizing the first one may be due to the process known as associative learning [26]. Egg parasitoids have developed strategies to find hosta and parasitize them, such as by detection of chemicals (semiochemicals) associated with the host or the damaged plant [41,42,43]. Associative learning may be related to the perception of chemical (semiochemical) and/or physical (visual or mechanical) stimuli of the first egg parasitized [44,45] and the parasitoid’s ability to locate, recognize and accept [46, 29, 26, 43] or reject other hosts [47]. Due to the small size of their hosts, female egg parasitoids have developed a capacity to respond to these stimuli to decrease recognition time and increase parasitism capacity in a shorter time [43]. This behaviour pattern was reported for *Anagrus pseudococci* Girault (Hymenoptera: Encyrtidae), which, after gaining experience, was more efficient in seeking hosts by moving faster and taking shorter time to handle additional hosts [48]. *Anaphes iole* Girault (Hymenoptera: Mymaridae) females, with previous oviposition experience were faster to parasitize *Lygus hesperus* Knight (Hemiptera: Lygaeidae) eggs than those without experience [47].

The fact that *C. noackae* females circled and touched the *T. peregrinus* eggs repeatedly with their antennae shows that this parasitoid needs morphological, chemical or sensory stimulation associated with its host to start oviposition [49]^.^ External investigation of the egg by virgin or mated *C. noackae* females, a process known as evaluation [26], allows the recognition of characteristics such as form, texture or movement of the host to verify its suitability for oviposition or to identify non-volatile chemicals released by other females during previous oviposition, thus avoiding superparasitism [50,47]. The time spent by *C. noackae* probing *T. peregrinus* with its antennae before inserting the ovipositor was similar to that reported for mated females, taking 35 seconds for this behavior [51]. However, the external evaluation time taken for a female to recognize and parasitize a host may decrease with successive egg handling experiences [52] and host age due to internal physical and chemical changes in the eggs [53,26] and with the recognition of host chemicals or lesions produced during ovipositor insertion [47]. The mean evaluation time of *Spartocera dentiventris* Berg (Hemiptera: Coreidae) eggs by the parasitoid *Gryon gallardoi* Brèthes (Hymenoptera: Scelionidae) increased with host age, from 78 to 201 seconds in 2 and 12-day-old eggs, respectively [53]. The previous parasitism experience of *A. iole* females decreased the evaluation time of subsequent hosts [47].

The oviposition of *C. noackae* immediately after contact with *T. peregrinus* eggs agrees with a previous report on this parasitoid, with an ovipositor insertion time of 2 to 10 minutes at 22 ºC for mated females [51]. Temperature and humidity may have affected the time taken for *C. noackae* to perforate *T. peregrinus* egg chorions and oviposit, since these factors affect host conditions and may modify female parasitoid behaviour [54,33], as reported for *Anaphes nitens* Girault and *Anaphes inexpectatus* Huber & Prinsloo (Hymenoptera: Mymaridae) on eggs of *Gonipterus platensis* Marelli (Coleoptera: Curculionidae), another exotic pest of eucalypts. The mating, groaning and oviposition of these parasitoids were more frequent between 10 and 25 ºC than at 5 ºC [55]. However, this varies among parasitoids, for example *A. delicatus* inserts its ovipositor in *Prokelisia marginata* Van Duzee (Homoptera: Delphacidae) eggs for 119 seconds (1.98 minutes) [37], a shorter time than that of *C. noackae* on bronze bug eggs. Variations in ovipositor insertion time among parasitoid species may be due to differences in host egg thickness, which affects cuticle perforation time [56].

Virgin or mated *C. noackae* females typically performed only one oviposition per *T. peregrinus* egg and rarely returned to the same egg; this behaviour was also observed if 20 *T. peregrinus* eggs were offered to females of this parasitoid in a glass vial (7.5 cm long x 2.5 cm wide) [24], while the frequency of return to and oviposition in the same egg was higher when 5 eggs were offered [51]. This behaviour may be associated with increased fitness and reproductive success [57]. Many females mark host eggs with chemical substances before leaving them [58,59]. This allows other females to recognize internal chemical changes of parasitized eggs [26], reducing the chances of superparasitism [60]. The non-recognition of co-specific markers and, consequently, the return of female parasitoids to previously oviposited eggs may be due to pheromone degradation and low oviposition experience of females [61,62].

*Cleruchoides noackae* oviposition soon after emergence is a common pattern for pro-ovigenic parasitoids [34], allowing them to produce offspring more quickly. However, parasitoids may exhibit time and/or egg deposition limitations on their hosts, which force them to develop strategies for host evaluation and oviposition to increase their fecundity and thus, their fitness [49].

The emergence of one *C. noackae* adult per *T. peregrinus* egg is a common pattern for this species; however, this may vary with host volume, species and quality and may influence parasitoid size [63,64]. The emergence of only one *C. noackae* individual per *T. peregrinus* egg can be explained by the fact that eggs of this pest are 0.48 mm long [65] and the *C. noackae*, 0.5 mm (after emerging) [22].

A sex ratio of 0.68 (in the progeny of mated females) is interesting because parasitoid females are responsible for parasitism, but the reproduction of *C. noackae* by arrhenotokous parthenogenesis is common among Mymaridae species [34]^.^ However, Hymenopteran females can adjust the sex ratio according to host size, age, quality, competition, temperature and oviposition sequence [66,67,68], to increase reproductive success.

The preference of *C. noackae* for insertion sites on the sides and operculum of *T. peregrinus* eggs does not appear to be due to the chorion thickness, which is approximately 0.44 mm all over the egg surface [65]. However, the operculum presents circular projections, probably aeromichromatic and smooth in texture in the outermost region of the egg, which may facilitate the penetration of the *C. noackae* ovipositor into the *T. peregrinus* egg [65]. In addition, this preference may be due to the fact that the *T. peregrinus* egg air layer has irregular columns from the endochorion up to the chorionic layer and the position of the *T. peregrinus* embryo inside the egg where the veal and wax coat is larger in the dorsal region of the egg than in the anterior (operculum) and lateral [65], which facilitates contact of the parasitoid ovipositor with the pest embryo.

**Conclusions**

Male and female *C. noackae* did not exhibit defined courtship behaviour and mated in less than one hour after making initial contact. The time taken to locate the first host, perform an external evaluation and insert the ovipositor (oviposition), as well as the percentage of inserted, parasitized and viable eggs were similar for virgin and mated *C. noackae* females. The sex ratio and the localization time of the other hosts was higher for mated and virgin females, respectively. The results obtained here contribute to improving strategies fpr *C. noackae* rearing and release in biological control programs for *T. peregrinus*.

**Acknowledgements**

To Proof-Reading-Services.com for revising and correcting the English language used in this manuscript.

**References**

1. Wingfield MJ, Slippers B, Hurley BP, Coutinho TA, Wingfield BD, Roux J. Eucalypt pests and diseases: growing threats to plantation productivity. South For. 2008; 70(2): 139-144.
2. Fernandes BF, Barcelos JAV, Andrade HB, Zanuncio JC. *Leptocybe invasa* (Hymenoptera: Eulophidae), an exotic pest of eucalyptus, in Minas Gerais State, Brazil. Fla Entomol. 2014; 97(2): 824-826.
3. Wilcken CF, Barbosa LR, Velozo SM, Becchi LK, Junqueira LR, de Sá LAN, et al. Biological control of *Thaumastocoris peregrinus* (Hemiptera: Thaumastocoridae) in eucalyptus plantations in Brazil: An Update. In: Mason PG, Gillespie DR, Vicent C, editors. Proceedings of the 5th International Symposium on Biological Control of Arthropods; 2017 Sept 11-15; Langkawi, Malaysia: CABI Wallingford UK, 2017; 6:105-107.
4. Wilcken CF, Soliman EP, de Sá LAN, Barbosa LR, Dias TKR, Ferreira Filho PJ, et al. Bronze bug *Thaumastocoris peregrinus* Carpintero and Dellapé (Hemiptera: Thaumastocoridae) on eucalyptus in Brazil and its distribution. J Plant Prot Res*.* 2010; 50(2): 201-205.
5. Almeida KECD, Silva JGSD, Silva IMDA, Costa ALD, Laia MLD. Ecophysiological analysis of *Eucalyptus camaldulensis* (Dehnh) submitted to attack from *Thaumastocoris peregrinus* (Carpintero & Dellape). Rev Árvore. 2018; 42(1): e420120.
6. Barbosa LR, Rodrigues AP, Souza LN, Foester LA, Souza AR, Castro BMC, et al. Development of *Cleruchoides noackae*, an egg-parasitoid of *Thaumastocoris peregrinus*, in eggs laid on different substrates, with different ages and post-cold storage. BioControl 2018; 63(2): 193-202.
7. Carpintero DL, Dellapé PM. A new species of *Thaumastocoris* Kirkaldy from Argentina (Heteroptera: Thaumastocoridae: Thaumastocorinae). Zootaxa 2006; 1228(1):61-68.
8. Noack A, Rose H. Life-history of *Thaumastocoris peregrinus* and *Thaumastocoris* sp. in the laboratory with some observations on behaviour. Gen App Entomol. 2007; 36:27-33.
9. Button G. *Thaumastocoris peregrinus*. Forestry facts [online]. Pietermaritzburg: NCT Forestry Co-operative Limited. Apr 2007; 63:16-17. Available from: <http://www.nctforest.com/upload/Publications/NV%202007%20April.pdf>.
10. Soliman EP, Wilcken CF, Pereira JM, Dias TK, Zaché B, Dal Pogetto MH, et al. Biology of *Thaumastocoris peregrinus* in different eucalyptus species and hybrids. Phytoparasitica 2012; 40(3): 223-230.
11. Lima ACV, Wilcken CF, Ferreira-Filho PJ, Serrão JE, Zanuncio JC. Intra-plant spatial distribution of *Thaumastocoris peregrinus* Carpintero & Dellapé (Hemiptera: Thaumastocoridae) on *Eucalyptus grandis* plants. Phytoparasitica 2016; 44(3): 411-418.
12. Wilcken CF, Barbosa LR, Soliman EP, Lima ACV, de Sá LAN, Lawson S. Percevejo-bronzeado-do-eucalipto, *Thaumastocoris peregrinus* Carpinteiro & Dellapé. In: Vilela EF, Zucchi RA, editors. Pragas Introduzidas No Brasil: Insetos e Ácaros, FEALQ; 2015. pp. 898-908.
13. Zanuncio JC, do Nascimento EC, Garcia JF. Major lepidopterous defoliators of eucalyptus, in the Southeast Brasil. For Ecol Manage. 1994; 65(1): 53-63.
14. Nadel RL, Noack AE. Current understanding of the biology of *Thaumastocoris peregrinus* in the quest for a management strategy. Int J Pest Manag. 2012; 58(3): 257-266.
15. Mascarin GM, Duarte VS, Brandão MM, Delalíbera JrI. Natural occurrence of *Zoophthora radicans* (Entomophthorales: Entomophthoraceae) on *Thaumastocoris peregrinus* (Heteroptera: Thaumastocoridae), an invasive pest recently found in Brazil. J Invertebr Pathol. 2012; 110(3): 401–404.
16. Lorencetti GAT, Potrich M, Mazaro SM, Lozano ER, Barbosa LR, Menezes MJS, et al. Eficiência de *Beauveria bassiana* Vuill. e *Isaria* sp. para o controle de *Thaumastocoris peregrinus* Carpintero & Dellapé (Hemiptera: Thaumastocoridae). Ciênc Florest.  2018; 28(1): 403-411.
17. Soliman EP, Castro BMC, Wilcken CF, Firmino AC, Pogetto MHFAD, Barbosa LR, Zanuncio JC. Susceptibility of *Thaumastocoris peregrinus* (Hemiptera: Thaumastocoridae), a eucalyptus pest, to entomopathogenic fungi. Sci Agric. 2019; 76(3): 255-260.
18. Barbosa LR, Santos F, Barddal HPO, Machado BO, Wilcken CF, Soliman EP. Predação de *Thaumastocoris peregrinus* por *Chrysoperla externa*. Comunicado técnico Embrapa Florestas. Nov 2010; 257(1): 1-4. Available from: <https://www.infoteca.cnptia.embrapa.br/bitstream/doc/870870/1/CT257.pdf>.
19. Souza GK, Pikart TG, Pikart FC, Serrão JE, Wilcken CF, Zanuncio JC. First record of a native heteropteran preying on the introduced eucalyptus pest, *Thaumastocoris peregrinus* (Hemiptera: Thaumastocoridae), in Brazil. Fla Entomol*.* 2012; 95(2): 517-520.
20. Dias TKR, Wilcken CF, Soliman EE, Barbosa LR, Serrão JE, Zanuncio JC. Predation of *Thaumastocoris peregrinus* (Hemiptera: Thaumastocoridae) by *Atopozelus opsimus* (Hemiptera: Reduviidae) in Brazil. Invertebrate Surviv J*.* 2014; 11(1): 224-227.
21. Cross D. The parasitoids of *Thaumastocoris* spp. in the Sydney region. M.Sc. Thesis, Department of Food and Natural Resources: University of Sydney, Australia. 2009.
22. Lin NQ, Huber JT, Salle JL. The australian genera of Mymaridae (Hymenoptera: Chalcidoidae). Zootaxa 2007; 1596(1): 1-111.
23. Barbosa LR, Rodrigues AP, Soler LS, Fernandes BV, Castro BMC, Wilcken CF, et al. Establishment in the field of *Cleruchoides noackae* (Hymenoptera: Mymaridae), an exotic egg parasitoid of *Thaumastocoris peregrinus* (Hemiptera: Thaumastocoridae). Fla Entomol*.* 2017; 100(2): 372-374.
24. Mutitu EK, Garnas JR, Hurley BP, Wingfield MJ, Harney M, Bush SJ, et al. Biology and rearing of *Cleruchoides noackae* (Hymenoptera: Mymaridae), an egg parasitoid for the biological control of *Thaumastocoris peregrinus* (Hemiptera: Thaumastocoridae). J Econ Entomol*.* 2013; 106(5): 1979-1985.
25. Souza AR, Candelaria MC, Barbosa LR, Wilcken CF, Campos JM, Serrão JE, et al. Longevity of *Cleruchoides noackae* (Hymenoptera: Mymaridae), an egg parasitoid of *Thaumastocoris peregrinus* (Hemiptera: Thaumastocoridae), with various honey concentrations and at several temperatures. Fla Entomol*.* 2016; 99(1): 33-37.
26. Vinson SB. The general host selection behaviour of parasitoid Hymenoptera and a comparison of initial strategies utilized by larvaphagous and oophagous species. Biol Control 1998; 11(2): 79-96.
27. Beserra E, Parra JRP. Comportamento de parasitismo de *Trichogramma atopovirilia* Oatman & Platner e *Trichogramma pretiosum* Riley (Hymenoptera, Trichogrammatidae) em posturas de *Spodoptera frugiperda* (J.E. Smith) (Lepidoptera, Noctuidae). Rev Bras Entomol*.* 2003; 47(2): 205-209.
28. Corbet SA. Insect chemosensory responses: a chemical legacy hypothesis. Ecol Entomol*.* 1985; 10(2): 143-153.
29. Nurindah BWC, Gordh G. Experience acquisition by *Trichogramma australicum* Girault (Hymenoptera: Trichogrammatidae). Aust J Entomol. 1999; 38(2): 115-119.
30. Barbosa LR, Santos F, Buhrer CB, Nichele LA, Wilcken CF, Soliman EP. Criação massal do percevejo bronzeado, *Thaumastocoris peregrinus*: Carpinteiro and Dellapé, 2006 (Hemiptera, Thaumastocoridae). Folheto, Embrapa Florestas. Jun 2016; 22p. Available from: <https://ainfo.cnptia.embrapa.br/digital/bitstream/item/145907/1/Criacao-massal-do-percevejo-bronzeado.pdf>.
31. Barbosa LR, Rodrigues AP, Nichele LA, Souza AR, [Becchi](https://www.embrapa.br/busca-de-publicacoes/-/publicacao/list/autoria/nome/luciane-katarine-becchi?p_auth=fwkPHRXq) LK, Wilcken CF. Orientações para a criação massal e liberação em campo de *Cleruchoides noackae* para controle biológico do percevejo bronzeado do eucalipto. Folheto, Embrapa Florestas. 2017; 23p. Available from: <https://ainfo.cnptia.embrapa.br/digital/bitstream/item/167917/1/Livro-TA-1393-completo.pdf>.
32. Rabeling C, Kronauer DJC. Thelytokous parthenogenesis in eusocial Hymenoptera. Annu Rev Entomol. 2013; (58): 273-292.
33. Gullan PJ, Cranston PS. Insetos - Fundamentos da Entomologia. 5th ed. Rio de Janeiro: Roca; 2017.
34. Jervis MA, Heimpel GE, Ferrns PN. Life-history strategies in parasitoid wasps: a comparative analysis of “ovigeny”. J Anim Ecol. 2001; 70(3): 442-458.
35. Jacobs DH, Neser S. *Thaumastocoris australicus* Kirkaldy (Heteroptera: Thaumastocoridae): a new insect arrival in South Africa, damaging to eucalyptus trees: research in action. S Afr J Sci. 2005; 101(5): 233-236.
36. Suzuki Y, Hiehata K. Mating systems and sex ratios in the egg parasitoids, *Trichogramma dendrolimi* and *T. papilionis* (Hymenoptera: Trichogrammatidae). Anim Behav. 1985; 33(4): 1223-1227.
37. Cronin JT, Strong DR. Biology of *Anagrus delicatus* (Hymenoptera: Mymaridae) an egg parasitoid of *Prokelisia marginata*. Ann Entomol Soc Am*.* 1990; 83(4): 846-854.
38. Usmani MK. Biological investigations on some species of *Anagrus* (Hymenoptera, Mymaridae), egg parasitoids of leafhoppers (Hemiptera). APCBEE Procedia 2012; (4): 1-5.
39. Davey KG. The female reproductive tract. In: Kerkut GA, Gilbert LI, editors. Comprehensive insect physiology, biochemistry and pharmacology. New York: Pergamon Press, Elmsford; 1985. pp. 15-36.
40. Neubaum DM, Wolfner MF. Wise, winsome, or weird? Mechanisms of sperm storage in female animals. Curr Top Dev Biol. 1999; (41): 67-97.
41. Fatouros NE, Dicke M, Mumm R, Meiners T, Hilker M. Foraging behavior of egg parasitoids exploiting chemical information. Behav Ecol. 2008; 19(3): 677–689.
42. Chiappini E, Salerno G, Berzolla A, Iacovone A, Reguzzi MC, Conti E. Role of volatile semiochemicals in host location by the egg parasitoid *Anagrus breviphragma*. Entomol Exp Appl. 2012; 144(3): 311-316.
43. Conti E, Collazza S. Chemical ecology of egg parasitoids associated with true bugs. Psyche J Entomol*.* 2012; (2012): 1-11.
44. Godfray HCJ. Parasitoids, behavioral and evolutionary ecology. Princeton University Press; 1994. 67: 1-473.
45. Dindo ML, Nakamura S. Oviposition strategies of tachinid parasitoids: two Exorista species as case studies. Int J Insect Sci. 2018; 10: 1-6.
46. Kaiser L, Pham-delegue MH, Masson C. Behavioural study of plasticity in host preferences of *Trichogramma maidis* (Hym.: Trichogrammatidae). Physiol Entomol. 1989; 14(1): 53-60.
47. Conti E, Jones WA, Bin F, Vinson SB. Oviposition behavior of *Anaphes iole*, an egg parasitoid of *Lygus hesperus* (Hymenoptera: Mymaridae; Heteroptera: Miridae). Ann Entomol Soc Am. 1997; 90(1): 91-101.
48. Islam KS. The influence of host and other environmental factors on the biology of the mealybug parasitoid *Anagyrus pseudococci* (Girault). PhD Thesis, University of London, United Kingdom; 1993.
49. Fellowes MDE, van Alphen JJM, Jervis MA. Foraging behavior. In: Jervis MA, editor. Insects as natural enemies. Springer, Dordrecht; 2005. pp. 1-72.
50. Vinson SB. Chemical signals used by parasitoids. Redia 1991; (74): 15-42.
51. Haas J, Barbosa LR, Potrich M, Lozano ER, Mazaro SM. Oviposition behavior of *Cleruchoides noackae* (Hymenoptera: Mymaridae) in the laboratory. Floresta e Ambiente 2018; 25(1): e00148115.
52. Wajnberg E. Analysis of variations of handling-time in *Trichogramma maidis*. Entomophaga 1989; 34(3): 397-407.
53. Rocha L, Redaelli L, Mendonça Jr MS. Oviposition behaviour of *Gryon gallardoi* (Hymenoptera, Scelionidae) on eggs of different ages of *Spartocera dentiventris* (Hemiptera, Coreidae). Iheringia Ser Zool. 2006; 96(3): 277-282.
54. Waage JK. Sib‐mating and sex ratio strategies in scelionid wasps. Ecol Entomol. 1982; 7(1): 103-112.
55. Valente C, Gonçalves CI, Reis A, Branco M. Pre-selection and biological potential of the egg parasitoid *Anaphes inexpectatus* for the control of the eucalyptus snout beetle, *Gonipterus platensis.* J Pest Sci. 2017; 90(3): 911-923.
56. Vinson SB. The behavior of parasitoids. In: Kerkut GA, Gilbert LI, editors. Comprehensive insect physiology, biochemistry and pharmacology. New York: Pergamon Press, Elmsford; 1985. pp. 417-469.
57. Moreira MD, Santos MCF, Beserra EB, Torres JB, Almeida RP. Parasitismo e superparasitismo de *Trichogramma pretiosum* Riley (Hymenoptera: Trichogrammatidae) em ovos de *Sitotroga cerealella* (Oliver) (Lepidoptera: Gelechiidae). Neotrop Entomol. 2009; 38(2): 237-242.
58. Strand MR, Ratner S, Vinson SB. Maternally induced host regulation by the egg parasitoid *Telenomus heliothidis*. Physiol Entomol. 1980; 8(4): 469-475.
59. Tanaka T. Calyx and venom fluids of *Apanteles kariyai* (Hymenoptera: Braconidae) as factors that prolong larval period of the host, *Pseudaletis separata* (Lepidoptera: Noctuidae). Ann Entomol Soc Am. 1987; 80(4): 530-533.
60. Darrouzet E, Bignon L, Chevrier C. Impact of mating status on egg-laying and superparasitism behaviour in a parasitoid wasp. Entomol Exp Appl. 2007; 123(3): 279-285.
61. Salt G. Experimental studies in insect parasitism. II. Superparasitism. P Roy Soc Lond B. 1934; 114(790): 455-476.
62. Okuda MS, Yeargan KV. Intra-and interspecific host discrimination in *Telenomus podisi* and *Trissolcus euschisti* (Hymenoptera: Scelionidae). Ann Entomol Soc Am*.* 1988; 81(6): 1017-1020.
63. Vinson SB. Comportamento de seleção hospedeira de parasitoides de ovos, com ênfase na família Trichogrammatidae. In: Parra JRP, Zucchi RA, editors. *Trichogramma* e o controle biológico aplicado. Piracicaba, FEALQ. 1997; pp. 67-119.
64. Bueno RCOF, Parra JRP, Bueno AF, Haddad M. Desempenho de tricogramatídeos como potenciais agentes de controle de *Pseudoplusia includens* Walker (Lepidoptera: Noctuidae). Neotrop Entomol. 2009; 38(3): 389-394.
65. Souza GK. Morfologia de ovos, glândulas salivares e sistemas digestivo e reprodutor de *Thaumastocoris peregrinus* (Hemiptera: Thaumastocoridae). M.Sc. Thesis. Entomology department: Federal University of Viçosa, Viçosa, Brazil; 2012.
66. Waage JK. Family planning in parasitoids: Adaptive patterns of progeny and sex allocation. In: Waage J, Greatheat D, editors. Insect Parasitoids. Academic Press; 1986. pp. 63-96.
67. King BH. Offspring sex ratios in parasitoid wasps. Q Rev Biol. 1987; 62(4): 367-396.
68. Van Baaren J, Nénon JP, Boivin G. Comparison of oviposition behavior of a solitary and a gregarious parasitoid (Hymenoptera: Mymaridae). J Insect Behav. 1995; 8(5): 671-686.

**Supporting information**

**S1_Table. Time period (mean ± standard error) of localization of the first egg, other eggs, insertion of ovipositor in minutes, and evaluation of the egg in seconds per *Cleruchoides noackae* (Hymenoptera: Mymaridae) female on *Thaumastocoris peregrinus* (Hemiptera: Thaumastocoridae) eggs.** Significant differences as determined by the unpaired t-test (a) or Mann-Whitney U test (b) are indicated with * (p<0.05) and non-significant differences with NS.

**S2_Table. Eggs inserted (%), parasitism (%), viability (%) and sex ratio (mean ± standard error) of virgin and mated *Cleruchoides noackae* (Hymenoptera: Mymaridae) females on *Thaumastocoris peregrinus* (Hemiptera: Thaumastocoridae) eggs during one hour of evaluation.** Significant differences as determined by the unpaired t-test (a) or Mann-Whitney U test (b) are indicated with * (p<0.05) and not significant differences with NS.

**S3_Table. Frequency (mean ± standard error) of the ovipositor insertion site per virgin or mated female of the parasitoid *Cleruchoides noackae* (Hymenoptera: Mymaridae) on *Thaumastocoris peregrinus* (Hemiptera: Thaumastocoridae) eggs.** Means followed by the same lowercase letter, per line or upper case, per column, do not differ by the two-way ANOVA (Tukey test, p < 0.05).

**S1_Dataset_Table. Dataset of time period of localization, insertion of ovipositor and evaluation of the *T. peregrinus* eggs per *Cleruchoides noackae* female.**

**S2_Dataset_Table. Dataset of eggs inserted, parasitism, viability and sex ratio of virgin and mated *C. noackae.***

**S3_Dataset_Table. Dataset of frequency of the ovipositor insertion site per virgin or mated female of the parasitoid *C. noackae.***
