# Supplementary material for: Oviposition behaviour of mated or unmated Cleruchoides noackae (Hymenoptera: Mymaridae)
Source: PLoS One. 2020 Oct 2;15(10):e0239285. doi: 10.1371/journal.pone.0239285 (PMC7531830; doi:10.1371/journal.pone.0239285)
Supplement: Attachment — Submitted filename: Response to Reviewers.docx [file pone.0239285.s006.docx]

Brazil, June, 2020

Dear Dr. Frank H. Koch,

Thank you for giving us the opportunity to improve and resubmit a revised version of our manuscript titled “Oviposition behaviour of mated or unmated *Cleruchoides noackae* (Hymenoptera: Mymaridae) females”, to PLOS ONE. We appreciate the time and effort that you and the reviewers have dedicated to providing your valuable feedback on our manuscript. We would like to thank for critical review of the manuscript, comments and helpful suggestions.

We have been able to incorporate changes to reflect most of the suggestions provided by the reviewers as you can see at “revised manuscript with track changes”. We reviewed the manuscript and incorporated suggestions of the reviewers. Following the recommendations of them, we highlight in the new version of the manuscript the new information generated in the present study about the oviposition behavior of *C. noackae* that was not covered in the articles of Mutitu et al (2013) and Haas et al (2018). As well as, our study present differences in the experimental methodology when compared with these published articles.

Below I explain in detail what the reviewers requested:

**Reviewer 1: found that the manuscript had some conceptual overlap with two previously published articles about the oviposition behavior of C. noackae: Mutitu et al. (2013) and Haas et al. (2018). Editor: These articles are cited in the manuscript, but it is important that you explain clearly how your study builds upon and adds to these previous publications.**

The reproductive behavior of *C. noackae* needed further studies, even with previously published articles because the time takes for males and female *C. noackae* to find each other after their emergence was unknown. This information is very important to known the optimal time that we need to wait to offer host eggs to copulated female, in order to avoiding the arrhenotokous parthenogenesis, described in this species. The articles of Mutitu (2013) and Hass (2018), do not show the time taken for *C. noackae* females to find other host eggs after parasitizing the first one, the percentage of *T. peregrinus* eggs that *C. noackae* female inserted the ovipositor and which were effectively parasitized in one hour, and the frequency (%) of ovipositor insertion site (operculum or opposite to the operculum) by *C. noackae* in the *T. peregrinus* eggs.

We adapted the methodology described by these authors to our conditions of mass rearing *C. noackae* in the laboratory of São Paulo State University, Brazil. We can observe differences, between the published articles by Mutitu et al. (2013) and Haas et al. (2018), mainly in the polystyrene vials where the parasitoid was reared, the temperature range used, the number of *T. peregrinus* eggs offered to the females, the age of *C. noackae* females used in the bioassays and the time of mating and oviposition behavior evaluation.

In our mating behavior experiment, we evaluated eleven parasitoid couples during one hour at 25 ºC, without offering hosts eggs. We used a transparent polystyrene tube (7.5 cm high x 3.0 cm in diameter) that we currently use in Brazil to rearing the parasitoid in the laboratory and for their release in the field. In the oviposition behavior experiment, we evaluated 10 virgin females and 10 mated females isolated, at most of 3 hours old, for one hour, at 25 ºC in a transparent polystyrene tube containing 10 *T. peregrinus* eggs per female.

**Reviewer 1 and 2: both reviewers commented on the quality of the written English. They identified a number of typographical and grammatical errors in the manuscript, as well as some places in where the language was unclear or ambiguous.**

All spelling, typographical and grammatical errors pointed out by the reviewers have been corrected.

**Reviewer 2: noted at least one technical error (with respect to comparing treatments in Table 2).**

This technical error have been corrected.

**Reviewer 1 and 2: Each reviewer attached an annotated copy of the manuscript with their specific comments and edits, which you should consider carefully.**

I have incorporate changes in the manuscript considering the suggestions and comments provided by the reviewers.

**Reviewer 2: expressed some concern regarding the volume of data, i.e., whether there are enough data to support robust conclusions. Editor: I believe the data are sufficient, but it would be good if you could provide readers with a comparison of the scope of your study (in terms of sample size, etc.) versus the scope of other, similar studies of oviposition behavior.**

We consider that our replications were similar to others published article of *C. noackae* behaviour. In the experiment of *C. noackae* mating behavior we did 11 replications, these are similar to the data volume in the published article of Mutitu et al. (2013), with n=10, and was higher to that Haas et al. (2013), with only n=5. In the oviposition behavior experiment, we evaluated 10 replications to virgin females and 10 for mated females, this was the same n evaluated by Mutitu et al., (2013). In Haas et al., (2018), they did 12 replications, but they only evaluated mated females.

**Review Comments to the Author**

**Reviewer # 1:**

**The paper is a useful study and contributes to our knowledge of the biology of *Cleruchoides noackae* as a key biocontrol agent for *Thaumastocoris peregrinus*. However, there is overlap with previously published papers on the oviposition behaviour of *C. noackae*, notably Mutitu et al 2013 and Haas et al 2018. It would be useful in the text to make specific reference to these papers and how the present study adds extra knowledge not covered by these studies to make the demarcation clear.**

Following your recommendation, in this document and on the revised manuscript, I highlighted the contributions of our research about the knowledge of the reproductive and oviposition behavior of *C. noackae* that were not addressed in the articles published by Mutitu et al 2013 and Haas et al 2018.

**The paper is technically sound and I believe the statistical analyses were appropriate for the studies carried out (although this is not my specialty).**

Thank you.

**One issue is that the English grammar needed correction which is to be expected from non-native English speaking authors. Edits are suggested in the attached Word document. Specific comments on the paper and edits that need to be addressed are included in the track changes document attached.**

All spelling, typographical and grammatical errors pointed out have been corrected and I have considered carefully all comments on the manuscript and edits.

**Once these changes are made I believe that the paper is suitable for publication.**

Thank you.

**Reviewer #2:**

**Dear Author, working on insect mating behavior is so valuable and interesting issue which can improve mass rearing techniques of natural enemies, but I think your manuscript cannot be published in this version due to some technical and linguistic problems.**

I apologize for the technical and linguistic problems. All spelling, grammatical and technical errors pointed out by the reviewers have been corrected.

I would like to thank again for the detailed revision of the manuscript and the recommendations that were very useful to improve it.

I am at your service if you have any questions.

Sincerely,

Luciane Katarine Becchi

Departamento of Plant Protection

Faculdade de Ciências Agronômicas, UNESP

18610-034, Botucatu, São Paulo, Brasil. E-mail: [becchiluciane@gmail.com](mailto:becchiluciane@gmail.com)

Tel: +55 44 996946892
