# Supplementary material for: Oviposition behaviour of mated or unmated Cleruchoides noackae (Hymenoptera: Mymaridae)
Source: PLoS One. 2020 Oct 2;15(10):e0239285. doi: 10.1371/journal.pone.0239285 (PMC7531830; doi:10.1371/journal.pone.0239285)
Supplement: Attachment — Submitted filename: Response to Reviewers.docx [file pone.0239285.s007.docx]

Brazil, August 2020

Dear Dr. Frank H. Koch,

We appreciate the time and effort that you and the reviewers have been dedicated to providing your valuable feedback on our manuscript. We would like to thank for critical review of the manuscript, comments and suggestions. Below I explain in detail what the reviewers and you requested, and my answers in blue phrases.

**Editor comments to the Author**

Your revised manuscript is much improved. There are still some editorial and grammatical issues to address, many of which I have documented in an annotated (tracked changes) version of the manuscript.

**Thank you. We have corrected the editing and grammar errors that were mentioned.**

Reviewer #3 also attached a more detailed set of comments for you to consider.

**I have incorporate changes in the manuscript considering the suggestions and comments provided by the reviewer and below I explain in detail.**

Reviewer #4 expressed concern about the suitability of the analysis given the limited number of replicates. As I mentioned in my comments about your initial submission, I believe the data are sufficient (marginally so), and I am mostly satisfied with your response to Reviewer #2 and myself regarding this issue ("We consider that our replications were similar to others published..."). However, now that two different reviewers have raised this issue, I believe it's important to integrate your response directly into the Discussion portion of the manuscript.	**As requested, I have incorporated my responses into the discussion (lines 327-332).**

Furthermore, I think you should elaborate on the consequences of your limited volume of data for interpretation. For instance, a small number of replicates meant that your standard errors were relatively large in some instances, which may have obscured actual differences between mated and unmated females that you would have detected with a larger number of replicates. Overall, I think you had some meaningful results regardless of their statistical significance, but it is always important to recognize and acknowledge the limitations and potential sources of error in an analysis. You will need to make this change, as well as all of the minor editorial/grammatical changes, for the manuscript to be suitable for publication.

**I recognize that the number of repetitions could be a limiting factor in our study. For this reason I added a sentence in the Discussion, that regarding the consequences of the limited data volume for the analysis and by the interpretation of our results. Additional information: In our study, the standardization of female and male *C. noackae* of age (<3 hours) from the same population and generation in the laboratory was the main limiting factor to have more repetitions.**

**A standardization difficulty is due to:**

**1) The sex ratio biased to the females (sex ratio> 0.65);**

**2) The restricted emergence period (the parasitoids emerge from 4:00AM) conditioned that the evaluation period was only in the morning;**

**3) The short life cycle of *C. noackae*, where the adults emerged up to three days; and**

**4) We had only two stereo microscopes available for the study of the parasitism behaviour.**

**Review Comments to the Author**

**Reviewer #4:**

This paper is a good contribute to improve the knowledge of the biology mainly with regard to reproductive biology of *Cleruchoides noackae* as a key biocontrol agent for *Thaumastocoris peregrinus.* Although most of the suggestions and corrections suggested by previous reviewers have been resolved by the authors, I believe that one of the most important aspects has not been improved; there is an unresolved technical problem related with design of the experiments (i.e., the number of replicates). The number of replicates presented in that study (N=11) is very low to support robustly any behavior related with reproductive biology, like courtship, mating or oviposition behaviors. In fact, in most of the analyzed parameters the standard error is very high in comparison to the average value, which does not offer security in the obtained values. The acceptable number of repetitions should be 3 to 4 times higher than what was considered.

**Thank you for your comments. I understand your restlessness by the less number of replicates. I don´t know how I can solve this technical problem. I recognize that the number of replicates can be a limiting factor in the analyses. However, as I mentioned in the text, the standardization of males and females of *C. noackae* of the same age (<3 hours), population and generation in the laboratory was a limiting factor to have more replicates. We consider that our replicates number were similar to another’s published articles of *C. noackae* behavior. In the experiment of *C. noackae* mating behavior we does 11 replicates, these are similar to the published article of Mutitu et al. (2013) (N=10), and was higher to that Haas et al. (2018), they only had 5 replicates. In our oviposition behavior experiment, we evaluated 10 virgin females and 10 mated females, this was the same number of replicates evaluated by Mutitu et al. (2013). In other hand, Haas et al., (2018), they presented 12 replicates, but they only evaluated mated females.**

Furthermore, there are some behaviors associated to parasitoids that are not observed by the authors, neither referred as associated with other species. This is the case, for example, of the probing behavior with the oviscapt (before the ovipositing behavior) that was observed associated to some parasitoids, such as the case of *Anagyrus vladimiri* Triapitsyn (= *Anagyrus* sp. nr. *pseudococci* (Girault)) (Bugila et al, 2014). This example indicates that the state of the art in this issue should have been further developed. For these reasons, I consider that this manuscript should not be published in this format.

**It was not possible to differentiate between the oviposition and probing behaviors in this species. After external evaluating, *C. noackae* females as soon as they inserted the ovipositor on *T. peregrinus* eggs kept their antennae parallel to the abdomen without wing movements and vertically lowered and raised the abdomen, characterizing "the oviposition". The females didn’t reject the eggs. We compared the oviposition behavior with other study with *C. noackae,* and this parasitoid is smallest than *Anagyrus* spp., that difficult the observations.**

**Reviewer #3:**

The study is valuable as it will contribute to improving laboratory rearing of the parasitoid *Cleruchoides noackae*. As a previous reviewer pointed out, there are language problems throughout the manuscript, which is understandable. However, in some places this forms a barrier that prevents the main ideas from being communicated fully. This could be corrected with the help of a copy editor, one with a scientific background may be helpful as they would better understand the technicalities of laboratory experiments. The references could be looked at to make sure that references more specific to the study are used.

**Thank you for your comments. Excuse me, and I'm sorry about the language problems. I have incorporated changes in the manuscript considering your valuable suggestions and comments.**

**Others suggestions and questions Reviewer #3:**

**Title**

“females” could be removed from the title. **I am in accord and remove it.**

**Abstract**

Line 27 – “...exotic Eucalyptus spp. pest.” change to “...exotic Eucalyptus spp. pest in Brazil”. **I am in accord and I have changed the sentence.**

**Introduction**

Second paragraph

- Line 54 doesn’t relate to the rest of the paper as a whole, it could be removed. **I have removed the sentence.**

Fourth paragraph

Line 67 consider replacing “endoparasitoid” with egg parasitoid. **I have replaced it.**

Fifth paragraph

- Line 74 this study was conducted in the laboratory so previously reported lab sex ratios could also be included. **OK. I have included.**
- Line 76, recommend including Mutitu et al. as a reference here as they have also published on longevity and food provision. **Ok. I have included.**
- General: Agree with the first reviewer that similar information has been published by Haas and Mutitu, this could be pointed out in this paragraph and explained that the current study expands on these tests. **I have pointed: lines 75 – 82.**

Sixth paragraph

- Knowing the ovipostion and mating behaviour would allow better lab rearing by improving the timing of mate and host provision. Please give details on how the field efficacy would be improved as once in the field egg and mate encounters can't be controlled**. I have changed the sentence because what I meant is that the parasitism efficacy in the field would be improved after release mated females.**
- Oviposition behaviour is well explained and in detail, however mating behaviour is not. This becomes important in the results where no defined mating behaviour is observed. More detailed information on what precisely was being observed for would enhance the paragraph. **Ok. I have explained: lines 84 - 87.**
- The aim is clearly defined. **Ok.**

**Materials and Methods**

Courtship and mating behaviour

- Expand on why one hour was chosen as the observation period. **We did evaluations in the laboratory that were used to set-up of the experiments. We observed that the one hour time, was an enough time to know if the parasitoid was capable of mating and leaving offspring. Considering that this species is a pro-ovigenic parasitoid.**
- Were the observations made at a particular time of day, i.e. did the authors suspect the wasps were diurnal? **Yes. The observations were done in the morning because this species present diurnal habits. They started to emerge at 4:00AM, and the evaluation was at 7:00 am.**

Oviposition behaviour

- Second paragraph:
  - Were there any eggs that did not hatch and did not have *Cleruchoides noackae* within after they were dissected? If there were, were they assigned as unparasitized or parasitized and the parasitoid did not successfully develop? **There were five eggs that were dissected and we found *T. peregrinus* nymphs. There were three eggs that did not have neither *C. noackae* nor *T. peregrinus* nymphs. It was assigned as unparasitized and infertile (non-viable) eggs.**
  - Was it possible to distinguish between a random first encounter with an egg or did the *C. noackae* show distinct search behaviour? **It was not possible to distinguish between a random first encounter with an egg.**
  - Viability might not be the right term to use, maybe successful parasitism attempts or something similar could be used instead. **Ok.**
  - The formulae provided work out percentages. **Ok.**
  - The last sentence should be moved more towards the beginning of the paragraph as it has information in it that is used in a formula. **Ok. I have moved.**

**Results**

Courtship and mating behaviour

- Line 167, usually the mean is written with the variance after it. **Ok. I have changed.**
- How many of the 11 female and male pairs mated? All the pair mated. **Yes, all the pair was mated.**

Oviposition behaviour

- Please clarify what “other eggs” refers to, are they the second eggs parasitized, the remaining 9 eggs or all the eggs parasitized within the hour? **I mean the subsequent 9 eggs.**
- Was the female that did not parasitize her eggs replaced with one that did, or was this replicate removed from the study? **The female wasn’t replaced or removed from the study.**
- Table 1: could consider using either minutes or seconds for the time periods. **Ok.**
- Line 197: how many females parasitized the same egg more than once? **Almost all females returned at most in one previously parasitized eggs.**
- Table 2: “sex ratio” could be changed to “sex ratio of offspring”. **Ok. I have changed.**
- Line 221: The significance of mating versus oviposition site preference, mating versus ovipositor insertion frequency and mated versus unmated oviposition site selection has not been introduced to the paper yet. This is further discussed in the Discussion section but should be in the Introduction and Materials and Methods as well. **Ok.**

**Discussion**

General

- There are instances where references that are more specific to *C. noackae* should be used above more general references, for example paragraph 1, line 237, references 32 and 33 can be replaced. Another example is in the same paragraph, line 239, reference 34. **Ok.**
- The authors have given the results of other studies in a few instances, for example line 271 “...evaluated in a plastic petri dish for 30 min taking 35 seconds for this behaviour at 22°C.” this could be changed to whether or not the results of the current differed from previous studies and why that is significant. **The example in the line 271 was similar to that our reported.**

First paragraph

- Line 239, *Thaumastocoris peregrinus* laying eggs in clusters is introduced in this paragraph, it may be possible to include this in the Introduction and explain why it is significant to the study in the Discussion. **Ok.**
- Line 238 - 240, *Cleruchoides noackae* would emerge at approximately the same time under laboratory conditions if the eggs were parasitized on the same day. This would not necessarily be the case for the field eggs unless they were laid on the same day and parasitized within the same time frame. Is there a reference that can support this or other information that may be missing that can be included to support the authors’ statement? **There is not a reference that can support it.**

Third paragraph

- The last two sentences are interesting but do not add extra value to the manuscript beyond what has already been discussed. Removing them can be considered. **I have removed.**

Fourth paragraph

- Line 285, do the studies referenced as numbers 54 and 33 refer to humidity, or humidity and temperature? **33 refers to temperature and 54 to the humidity and temperature.**
- Line 289, there are two separate ideas in this paragraph, the first that temperature and humidity play a role in the time taken for *C. noackae* to penetrate the chorion. The second that the time taken to oviposit is a function of the thickness of the chorion and that this is why different species take different times to oviposit. Although temperature, humidity and chorion thickness would all play a role in oviposition time, consider either breaking these ideas into different paragraphs or rewording the original paragraph so that the main message comes across clearly. **Ok. I have breaking these ideas into different paragraphs.**

Fifth paragraph

- Line 295, the number of times a female returned to the same egg could be included in the Results section, it would be interesting to have more detailed information on how frequently this occurs. **In each repetition, a female returned at most in one previously parasitized egg.**
- Line 300, “Many females...” could be changed to be more specific to which females mark host eggs. **Ok.**
- Line 303, I am not sure about the use of co-specific as a term here as there were no other females in the vials, please make sure that this use is correct. **Ok.**
- Line 302, a previous paragraph explained that the decrease in time taken to oviposit in the “other eggs” was due to associative learning. This paragraph says that ovipositing into the same egg is due to poor oviposition experience (poor associative learning?) Did the females that oviposited into the same egg take longer to parasitize the other eggs? I.e. is there an indication that they are poor learners? **The time to find subsequent hosts eggs by *C. noackae* was reduced due to associative learning, not the time of oviposition. A female returned at most in one previously parasitized egg of the ten offered in one hour.**

Sixth paragraph

- The main idea of this paragraph is not coming through clearly, this could be fixed by either rewording it or expanding on how time or egg laying limitations change host evaluation behaviours and how that relates to *C. noackae*. **Ok.**

Seventh paragraph

- Consider rephrasing the sentence starting in line 313, it implies that if the *T. peregrinus* eggs were larger, there would be more than one *C. noackae* within them. **Ok.**

**Conclusions:**

- The conclusion section is clear. **Ok.**

**I apologize for the technical and linguistic problems. All spelling, grammatical and technical errors pointed out by the editor and reviewers have been corrected. I would like to thank again for the detailed revision of the manuscript and the recommendations that were very useful to improve it.**

**I am at your service if you have any questions.**

**Sincerely,**

**Luciane Katarine Becchi**

**Departamento of Plant Protection**

**Faculdade de Ciências Agronômicas, UNESP**

**18610-034, Botucatu, São Paulo, Brasil. E-mail:** [**becchiluciane@gmail.com**](mailto:becchiluciane@gmail.com)

**Tel: +55 44 996946892**
